# The maternal origin of indigenous domestic chicken from the Middle East, the north and the horn of Africa

**DOI:** 10.1186/s12863-020-0830-0

**Published:** 2020-03-14

**Authors:** Ahmed S. Al-Jumaili, Selma Farah Boudali, Adebabay Kebede, Sahar A. Al-Bayatti, Abdulamir A. Essa, Abulgasim Ahbara, Riyadh S. Aljumaah, Raed M. Alatiyat, Joram M. Mwacharo, Gro Bjørnstad, Arifa N. Naqvi, Semir Bechir Suheil Gaouar, Olivier Hanotte

**Affiliations:** 1 School of Life Sciences, the University of Nottingham, University Park, Nottingham, NG7 2RD UK; 2 University of Anbar, Ministry of Higher Education and Scientific Research, Anbar, Iraq; 3 Laboratoire de Génétique Moléculaire et Cellulaire, Université des Sciences et de la Technologie d’Oran Mohamed Boudiaf, USTO-MB, BP 1505, El M’naouer, Oran, 31000 Algérie; 4 Amhara Regional Agricultural Research Institute (ARARI), P.O. Box:527 Code 100, Bahir Dar, Ethiopia; 5 LiveGene, International Livestock Research Institute (ILRI), P. O. 5689, Addis Ababa, Ethiopia; 6 Animal Sources Department, Directorate of Animal Resources, Ministry of Agriculture, Baghdad, Iraq; 7 Animal Biotechnology, Animal Science Department, College of Food and Agriculture, King Saud University, P.O.Box 246, Riyadh, 11451 Kingdom of Saudi Arabia; 8 Genetics and Biotechnology, Animal Science Department, Agriculture Faculty, Mutah University, Karak, Jordan; 9 Small Ruminant Genetics and Genomics Group, International Centre for Agricultural Research in the Dry Areas (ICARDA), P.O. Box 5689, ILRI-Ethiopia Campus, Addis Ababa, Ethiopia; 10 Department of Forensic Sciences, Oslo University Hospital, Oslo, Norway; 11 Faculty of Life Sciences, Karakorum International University, Gilgit Baltistan, Pakistan; 12 Physiopathology and Biochemical of Nutrition (PpBioNut), University of Tlemcen, Tlemcen, Algeria

**Keywords:** Domestic chicken, Dispersal routes, Genetic diversity, Middle East, Africa

## Abstract

**Background:**

Indigenous domestic chicken represents a major source of protein for agricultural communities around the world. In the Middle East and Africa, they are adapted to hot dry and semi-dry areas, in contrast to their wild ancestor, the Red junglefowl, which lives in humid and sub-humid tropical areas. Indigenous populations are declining following increased demand for poultry meat and eggs, favouring the more productive exotic commercial breeds. In this paper, using the *D*-loop of mitochondrial DNA as a maternally inherited genetic marker, we address the question of the origin and dispersal routes of domestic chicken of the Middle East (Iraq and Saudi Arabia), the northern part of the African continent (Algeria and Libya) and the Horn of Africa (Ethiopia).

**Results:**

The analysis of the mtDNA *D*-loop of 706 chicken samples from Iraq (*n* = 107), Saudi Arabia (*n* = 185), Algeria (*n* = 88), Libya (*n* = 23), Ethiopia (*n* = 211) and Pakistan (*n* = 92) show the presence of five haplogroups (A, B, C, D and E), suggesting more than one maternal origin for the studied populations. Haplogroup E, which occurred in 625 samples, was the most frequent in all countries. This haplogroup most likely originates from the Indian subcontinent and probably migrated following a terrestrial route to these different countries. Haplotypes belonging to haplogroup D were present in all countries except Algeria and Libya, it is likely a legacy of the Indian Ocean maritime trading network. Haplogroup A was present in all countries and may be of commercial origin. Haplogroup B was found only in Ethiopia. Haplogroup C was only detected in the South-Western region of Saudi Arabia and in Ethiopia.

**Conclusion:**

The results support a major influence of the Indian subcontinent on the maternal diversity of the today’s chicken populations examined here. Most of the diversity occurs within rather than between populations. This lack of phylogeographic signal agrees with both ancient and more recent trading networks having shaped the modern-day diversity of indigenous chicken across populations and countries.

## Background

Undoubtedly, village chickens are a valuable genetic resource for the countries around the world due to their adaptation to the local environment, including their higher resistance against endemic diseases. They supply high-quality protein and represent a major source of income to poor communities. Therefore, they contribute greatly to food security, poverty alleviation and management of natural resources [1]. The main production system of indigenous chicken is scavenging or semi-scavenging, which relies on a low level of inputs. This system makes up to 80% of the poultry stocks in the developing countries of Asia and Africa [2].

The Red junglefowl is the main ancestor of the domestic chicken [3]. Its natural habitat is the sub-humid and humid tropical areas in South and South-East Asia. In contrast to the wild ancestor, village chickens have adapted very effectively to a diversity of environments including the arid and semi-arid areas. They show extensive morphological diversity that may be connected to the adaptation to such hot and dry environments, including the naked-neck phenotype, small body size and frizzled plumage [4–6]. Recent genome studies have revealed candidate regions under positive selection which may be related to environmental adaptation in this species [7].

MtDNA analysis has been used intensively to unravel the history of domestic chickens [8]. Analysis of the mitochondrial DNA genome allows us to identify the wild ancestor(s) and the maternal lines that have contributed to a breed or population [9, 10]. Furthermore, such genetic marker can offer valuable information concerning the human-mediated dispersal of the species out of the domestication centres [10]. Finally, mtDNA characterisation of diversity may help the establishment of effective management practices and sustainable strategies for the conservation of diversity.

This study aims to unravel the history and diversity of indigenous chicken from the Middle East, Northern and the Horn of Africa. It includes chicken from (i) Pakistan, a putative ancient centre of origin for domestic chicken in the northern part of the Indian subcontinent [11], (ii) Iraq, with its ancient Mesopotamian civilizations in contact with those of the Indus Valley, (iii) Saudi Arabia on the route to the African continent, (iv) the Horn of Africa, represented here by Ethiopia, where the oldest osteological evidence of domestic chicken for Africa have been found, dated from pre-Aksumite time [12], and (v) Algeria and Libya, two countries bordering the Mediterranean Sea, which witnessed ancient Phoenician, Greek and Roman terrestrial and maritime trading networks between North Africa, the Near East and Europe [13]. A total of 706 mtDNA *D*-loop sequences and Asian haplotypes of reference from Liu et al. [14] was analysed.

## Results

### *D*-loop haplotype variation and genetic diversity

Eighty-eight haplotypes defined by 63 polymorphic sites were identified in the 706 sequences (Additional file 1: Fig. S1). Each haplotype was abbreviated with the letter H followed by a number e.g. H_1, H_2, H_3 etc. The most frequent haplotype (41%) was H_3 (291 sequences out of 706). H_3 includes 92 (50%), 60 (28%), 58 (54%), 55 (63%), 14 (15%), 12 (52%) of Saudi, Ethiopian, Iraqi, Algerian, Pakistani and Libyan sequences respectively. The next commonest haplotypes were H_23 and H_4, 7.6 and 6% of all the sequences, respectively. At a country level, other common haplotypes included H_23 present only in Ethiopian samples (*n* = 54, 26%) and H_4 present only in Pakistani chicken (*n* = 43, 47%).

The Central region of Iraq showed a highly significant (*P* ≤ 0.001) and significant (*P* ≤ 0.05) levels of genetic diversity compared to the other regions (North-East and South), with haplotype diversities of 0.725 ± 0.053 (Baghdad) and 0.712 ± 0.105 (Karbala), but 0.438 ± 0.121 for Misan in the South-East, and 0.182 ± 0.144 for Basra in the South (Table 1 and Additional file 1: Table S2a). The same is applicable for the nucleotide diversity, which shows a highly significant (*P* ≤ 0.001) and significant (*P* ≤ 0.05) level of diversity in the Central region of the country (Additional file 1: Table S2b).

**Table 1:** Location, sample size and genetic diversity of the samples included in this study

Country/population	N	S	H	Hd (SD)	π (SD)	K
**Iraqi populations** **North-East**						
Sulimania [1]	9	0	1	0 (0)	0 (0)	0
**Central**						
Baghdad	51	18	12	0.725 (0.053)	0.0080 (0.0009)	3.167
Karbala	12	5	4	0.712 (0.105)	0.0043 (0.0010)	1.697
Central [2]	63	20	14	0.736 (0.051)	0.0075 (0.0008)	2.988
**South**						
Basra	11	1	2	0.182 (0.144)	0.0005 (0.0003)	0.182
Misan	24	11	5	0.438 (0.121)	0.0034 (0.0015)	1.359
South [3]	35	12	6	0.361 (0.103)	0.0025 (0.0011)	1.002
**Total**	107	22	18	0.686 (0.047)	0.0067 (0.0007)	2.652
**Algerian populations** **North-West**						
Mascara	20	3	4	0.363 (0.131)	0.0010 (0.0004)	0.389
Oran	17	5	6	0.706 (0.106)	0.0022 (0.0005)	0.882
Tiaret	11	2	2	0.182 (0.144)	0.0009 (0.0007)	0.364
Tlemcen	18	15	8	0.797 (0.088)	0.0056 (0.0021)	2.242
North-West [1]	66	19	12	0.569 (0.072)	0.0025 (0.0007)	1.030
**Central**						
Adrar [2]	22	5	4	0.654 (0.085)	0.0035 (0.0006)	1.403
**Total**	88	20	13	0.597 (0.060)	0.0028 (0.0006)	1.145
**Ethiopian populations** **North**						
Mihquan	10	9	3	0.644 (0.101)	0.0054 (0.0031)	2.156
Meseret	10	11	6	0.867 (0.085)	0.0072 (0.0026)	2.844
North [1]	20	14	7	0.742 (0.071)	0.0061 (0.0021)	2.447
**Central-East**						
Adane	10	11	5	0.800 (0.100)	0.0082 (0.0029)	3.244
Arabo	10	4	5	0.844 (0.080)	0.0033 (0.0006)	1.289
Horro	30	10	5	0.644 (0.081)	0.0068 (0.0017)	2.699
Jarso	14	0	1	0 (0)	0 (0)	0
Midir	10	2	3	0.600 (0.131)	0.0017 (0.0005)	0.667
Negasi_Amba	10	1	2	0.467 (0.132)	0.0012 (0.0003)	0.467
Central-East [2]	84	20	10	0.774 (0.027)	0.0092 (0.0010)	3.674
**West**						
Ashuda	10	1	2	0.356 (0.159)	0.0009 (0.0004)	0.356
Amshi	10	1	2	0.533 (0.095)	0.0013 (0.0002)	0.533
Batambie	8	1	2	0.250 (0.180)	0.0006 (0.0004)	0.250
Dikuli	10	2	3	0.600 (0.131)	0.0017 (0.0005)	0.667
Gafera	10	1	2	0.467 (0.132)	0.0012 (0.0003)	0.467
Surta	9	1	2	0.222 (0.166)	0.0006 (0.0004)	0.222
Tzion_Teguaz	10	3	3	0.600 (0.131)	0.0024 (0.0009)	0.933
West [3]	67	5	8	0.707 (0.044)	0.0025 (0.0002)	1.000
**South**						
Girissa	10	4	5	0.667 (0.163)	0.0027 (0.0008)	1.067
Kumato	10	10	5	0.756 (0.130)	0.0082 (0.0030)	3.244
Loya	10	8	5	0.756 (0.130)	0.0089 (0.0020)	3.533
Shubi_Gemo	10	14	6	0.911 (0.062)	0.0145 (0.0020)	5.756
South [4]	40	23	19	0.929 (0.021)	0.0150 (0.0006)	5.964
**Total**	211	33	36	0.840 (0.016)	0.0094 (0.0007)	3.732
**Saudi Arabia**						
East [1]	45	18	15	0.856 (0.044)	0.0042 (0.0008)	1.697
Central [2]	43	20	11	0.631 (0.084)	0.0037 (0.0010)	1.499
West [3]	97	23	16	0.702 (0.041)	0.0041 (0.0006)	1.658
**Total**	185	34	26	0.727 (0.033)	0.0041 (0.0005)	1.633
**Other populations**						
**Libya**	23	14	10	0.731 (0.099)	0.0054 (0.0018)	2.142
**Pakistan**	92	24	19	0.756 (0.043)	0.0088 (0.001)	3.503
**Total**						
**All the samples included in this study**	706	55	88	0.815 (0.014)	0.0077 (0.0004)	3.058

N = number of samples, S = segregating sites, H = Number of haplotypes, Hd (SD) = haplotype diversity (standard deviation), π (SD) = nucleotide diversity (standard deviation) and K = average number of nucleotide differences

For the Algerian populations, Adrar, Oran and Tlemcen show higher haplotypes (0.654 ± 0.085, 0.706 ± 0.106 and 0.797 ± 0.088) and nucleotides diversities (0.0035 ± 0.0006, 0.0022 ± 0.0005 and 0.00565 ± 0.0021) compared to the haplotypes (0.363 ± 0.131 and 0.182 ± 0.144) and nucleotides (0.001 ± 0.0004 and 0.0009 ± 0.0007) diversities for Mascara and Tiaret, respectively. Significant (*P* ≤ 0.05) haplotypes diversity differences found between Tlemcen and Tiaret, Tlemcen and Mascara, Adrar and Tiaret and Oran and Tiaret (Additional file 1: Table S3a) populations. On the other side, only two significant differences (*P* ≤ 0.001 and *P* ≤ 0.05) for nucleotide variation between Mascara and Tlemcen on the first level, and between Tiaret and Tlemcen on the second level (Additional file 1: Table S3b) are observed.

In Ethiopia, different populations show different levels of diversity, with a highly significant (*P* ≤ 0.001) and significant (*P* ≤ 0.05) haplotypes diversities for Adane, Arabo, Kumato, Loya, Meseret and Shubi (Additional file 1: Table S4a), and with the nucleotide diversities for the same populations ranging between 0.0033–0.0145, with a highly significant (*P* ≤ 0.001) or significant (*P* ≤ 0.05) differences among populations (Additional file 1: Table S4b). At a regional level, the North, Central-East and West regions do not show significant differences in their diversities, but the South region has the highest level of genetic variation compared to the other regions.

The Saudi Arabian regions also show different significant levels of diversity (Additional file 1: S5a), with the highest haplotype diversity in the East on the shores of the Arabian Gulf (0.856 ± 0.044), then the West on the shores of the Red Sea (0.702 ± 0.041), while the lowest was in the Central region (0.631 ± 0.084). No significant differences are observed in Saudi Arabia for the nucleotide variation (Additional file 1: Table S5b). Libya and Pakistan show high diversity levels for both haplotype (0.731 ± 0.099 and 0.756 ± 0.043 respectively) and nucleotide diversity (0.0054 ± 0.0018 and 0.0088 ± 0.001, respectively).

At the country level, the highest haplotype diversity was present in Ethiopia and the lowest in Algeria. An intermediate level of haplotype variation was observed in Iraq, Libya, Pakistan and Saudi Arabia.

### Phylogeographic analysis

**Fig. 1:**
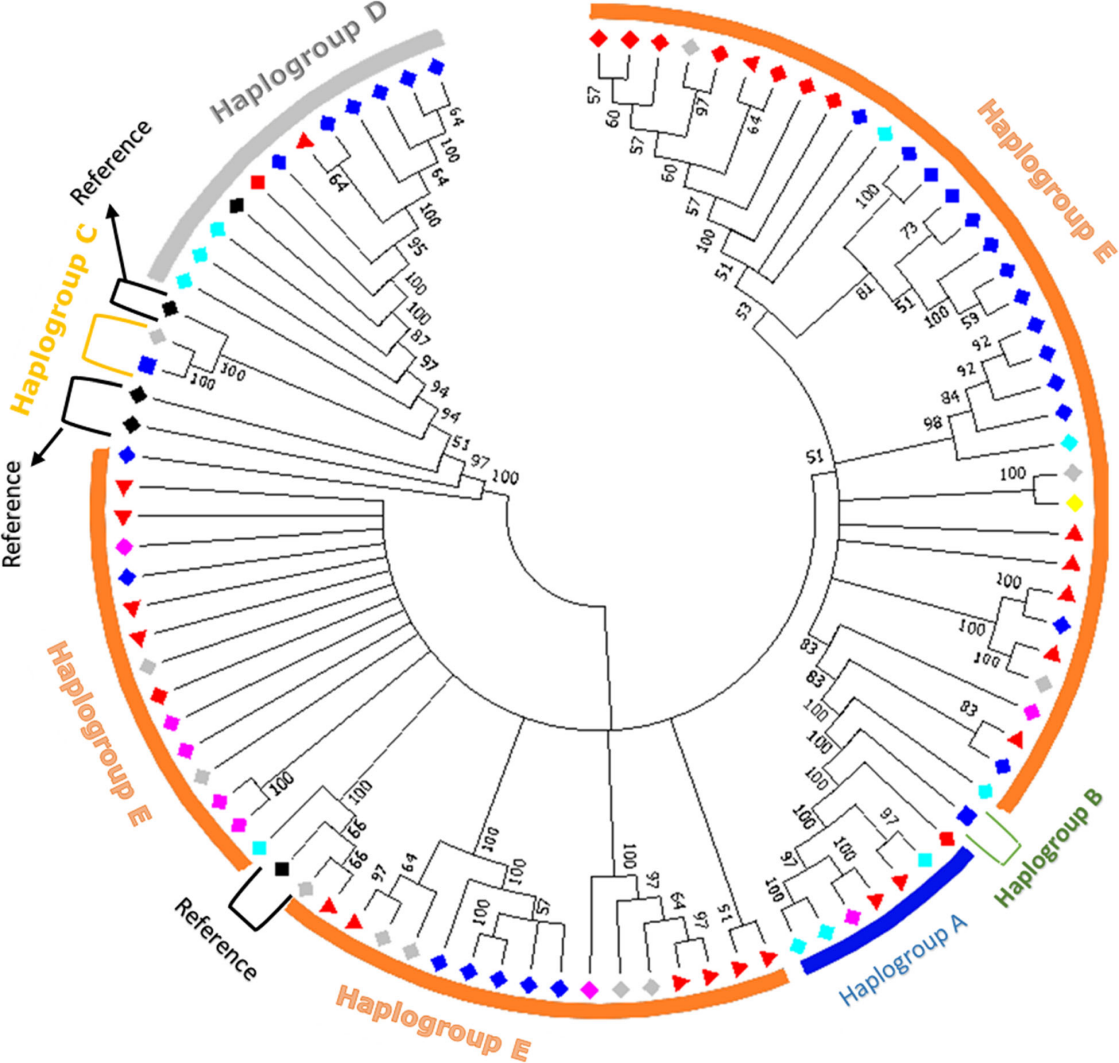
Maximum likelihood tree for the 88 haplotypes and references.  = Iraqi haplotypes,  = Ethiopian haplotypes,  = Algerian haplotypes,  = Saudi haplotypes,  = Pakistani haplotypes,  = Libyan haplotypes,  = Ethiopian haplotype with reference,  = References,  = common haplotypes among countries. Numbers on nodes represent bootstrap values

**Fig. 2:**
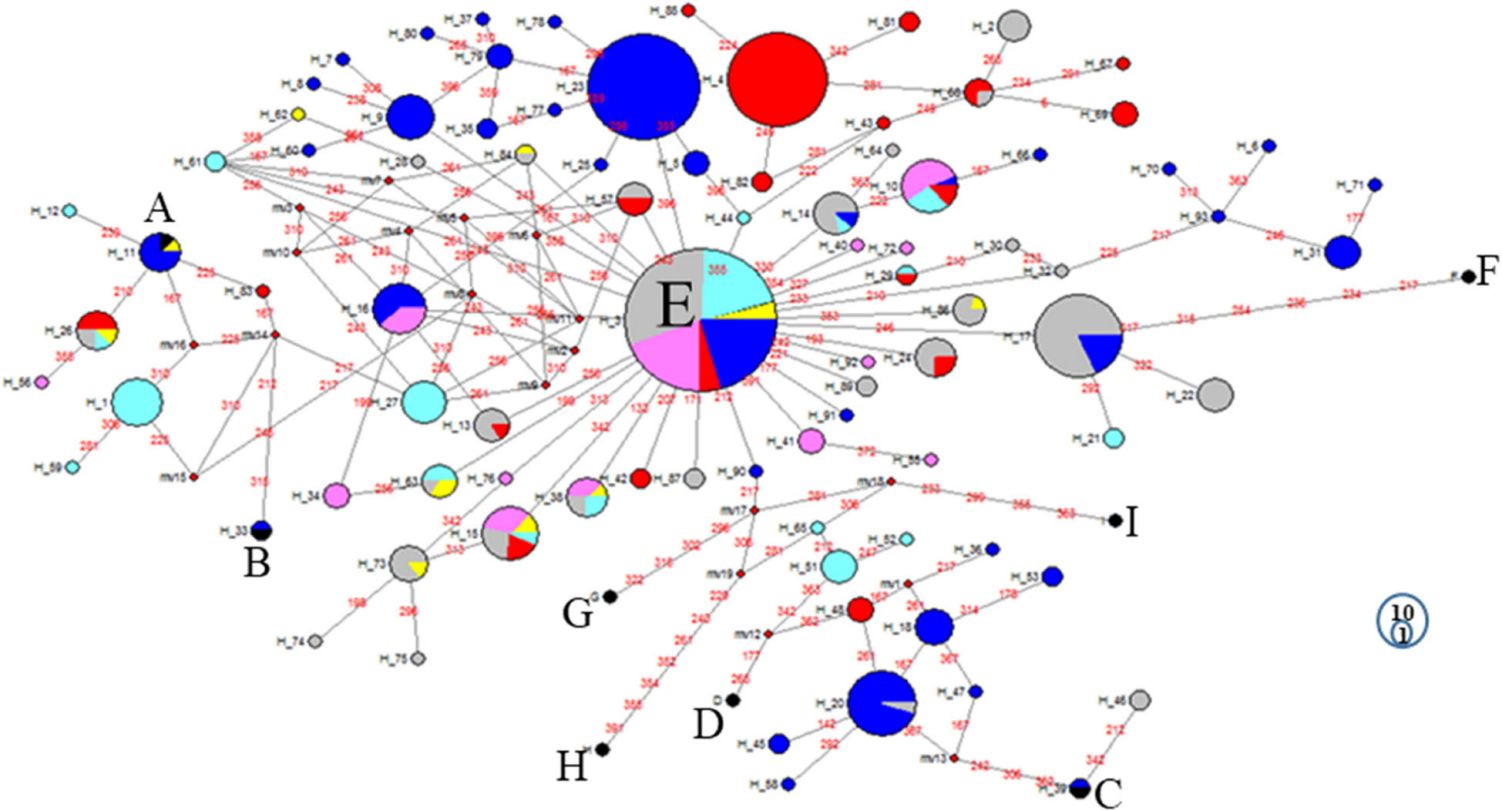
Median-Joining network for Algeria, Ethiopia, Iraq, Libya, Pakistan and Saudi Arabia haplotypes. The black circles refer to the reference haplotypes. Pink = Algerian haplotypes, Blue = Ethiopian haplotypes, Cyan = Iraqi haplotypes, Yellow = Libyan haplotypes, Red = Pakistani haplotypes and Grey = Saudi Arabian haplotypes. The numbers on the branch indicate the position of the mutations, the circles are proportional to the numbers of haplotypes

Reference haplotypes from Liu et al. [14] were used to name the haplogroups accordingly to their nomenclatures (a, B, C, D, E, F, G, H and I). A maximum likelihood tree for the 88 haplotypes was constructed to assess the genetic relationships among them (Fig. 1). Most branches included show high confidence (bootstrap) relationship values ranging between 70 and 100, and suggest the presence of five haplogroups (a, B, C, D, and E). The haplotypes were classified in different haplogroups following the results of the tree and network for all the countries together, and an individual network for each country separately (Figs. 1, 2, Additional file 1: Fig. S2, Additional file 1: Fig. S3, Additional file 1: Fig. S4, Additional file 1: Fig. S5, Additional file 1: Fig. S6 and Additional file 1: Fig. S7). The frequency of each haplogroup in each country is indicated in Table 2. Haplogroup E is the most frequent one (625 individuals out of 706) followed by D (*n* = 46), a (*n* = 31), C (n = 3) and B (*n* = 1). Haplogroup a is represented by 7 haplotypes, B by one, C by two and haplogroup D by 11 haplotypes

**Table 2:** Distribution of haplotypes across haplogroups and countries

Haplogroup/Haplotypes	Algeria	Ethiopia	Iraq	Libya	Pakistan	Saudi Arabia
**Haplogroup A**						
H_1			12			
H_11		6		1		
H_12			1			
H_26			1	1	4	2
H_56	1					
H_59			1			
H_83					1	
**Haplogroup B**						
H_33		1				
**Haplogroup C**						
H_39		1				
H_46						2
**Haplogroup D**						
H_18		7				
H_20		20				1
H_36		1				
H_45		2				
H_47		1				
H_48					3	
H_51			6			
H_52			1			
H_53		2				
H_58		1				
H_65			1			
**Haplogroup E**						
H_2						5
H_3	55	60	58	12	14	92
H_4					43	
H_5		3				
H_6		1				
H_7		1				
H_8		1				
H_9		10				
H_10	8	1	4		2	
H_13					1	5
H_14		1	1			7
H_15	5		1	2	3	4
H_16	5	8				
H_17		6				27
H_21			2			
H_22						6
H_23		54				
H_24					2	6
H_25		1				
H_27			9			
H_28						1
H_29			1		1	
H_30						1
H_31		6				
H_32						1
H_34	3					
H_35		2				
H_36						
H_37		1				
H_38	3		2	1		2
H_40	1					
H_41	3					
H_42					2	
H_43					1	
H_44			1			
H_55	1					
H_57					3	3
H_60		1				
H_61			2			
H_62				1		
H_63			3	2		1
H_64						1
H_66		1				
H_67					1	
H_68					3	1
H_69					3	
H_70		1				
H_71		1				
H_72	1					
H_73				1		6
H_74						1
H_75						1
H_76	1					
H_77		1				
H_78		1				
H_79		3				
H_80		1				
H_81					2	
H_82					2	
H_84				1		1
H_85					1	
H_86				1		4
H_87						2
H_89						2
H_90		1				
H_91		1				
H_92	1					
H_93		1				

Haplogroup E is represented by different numbers of individuals and proportions in the six countries. It is present in 87 (99%), 169 (80%), 84 (79%), 21 (91%), 84 (91%) and 180 (97%) birds out of 625 for Algeria, Ethiopia, Iraq, Libya, Pakistan and Saudi Arabia, respectively. Haplogroup A is found in 31 samples (all countries). Haplogroup B and C were only found in Ethiopia and Saudi Arabia. One haplotype from Ethiopia was identical to the reference haplotype B, while C was identified as one Ethiopian haplotype and one Saudi haplotype (represented by two samples from the South-West region). Haplotypes belonging to haplogroup D were present in all countries except Algeria and Libya. This haplogroup was represented by eleven haplotypes beside the reference one. It includes 34 (16%), 8 (7%), 3 (7%) and 1 (0.5%) samples, out of 46, for Ethiopia, Iraq, Pakistan and Saudi Arabia respectively.

Network analysis allows further visualisation of the diversity of the haplotypes and their relationships. The most frequent haplotypes (H_3, H_4 and H_23), belong to haplogroup E (Fig. 2). The first (H_3) is identical to the haplotype reference, H_4 is different from the previous one by 4 mutations only, while only a single mutation separates H_23 from H_3. The links between haplogroup E and the other haplogroups are well resolved. Haplogroup E is separated from haplogroup D by several mutations with three median vectors (mv), while C is connected to E by 12 mutations and 5 mv. The same situation is applicable to other clusters except for haplogroup F which was connected to E by 7 mutations without median vectors. The presence of median vectors could be attributed to un-sampled haplotypes, e.g. haplotypes that have not been introduced to the geographical area or haplotypes that became extinct [15]. Haplotype H_3 (haplogroup E) at the centre of a star-like pattern is likely the ancestral haplotype for this haplogroup.

Both phylogenetic tree and network (Additional file 1: Fig. S8 & S9) for the downloaded sequences showed similar results compared to phylogenies of the collected samples. These results include the same haplogroups (A, B, C, D and E), and similar pattern of haplotypes distribution.

### Analysis of molecular variance (AMOVA)

Both maternal genetic differentiation within and among populations were assessed for the samples included in this study (Table 3). The data were examined at three levels, among groups (regions within country) of populations, among populations within groups and among individuals within populations. The results show that the highest percentage of variation for Iraq and Algeria is found among individuals, 66.33 and 94.41% respectively, within populations. The percentage of differentiation among groups of Iraqi populations is higher than the one observed in Algeria, 28.89 and 5.03% respectively. In Ethiopia, the percentage of variation for both populations within groups (45.84%) and among individuals within populations (42.87%) are relatively large, with lower variation among groups (11.29%). The three regions of Saudi Arabia show most of the variation (96.66%) among individuals within regions, and hardly any genetic differentiation among regions (3.34%). In general, when we compare all the countries (Algeria, Ethiopia, Iraq, Libya, Pakistan and Saudi Arabia), the highest variation is found among individuals within populations (74.12%), then among populations within countries (17.01%).

**Table 3:** Analysis of Molecular Variance (AMOVA)

Grouping	Source of variation	Degrees of freedom	Variance components	Percentage of variation
**Iraq**	Among groups	2	0.44860 Va	28.89
	Populations within groups	2	0.07417 Vb	4.78
	Within populations	102	1.02984 Vc	66.33
	Total	106	1.55261	
**Algeria**	Among groups	1	0.02977 Va	5.03
	Populations within groups	3	0.00331 Vb	0.56
	Within populations	83	0.55855 Vc	94.41
	Total	87	0.59163	
**Ethiopia**	Among groups	3	0.22407 Va	11.29
	Populations within groups	15	0.91011 Vb	45.84
	Within populations	192	0.85103 Vc	42.87
	Total	210	1.98520	
**Saudi Arabia**	Among regions	2	0.02819 Va	3.34
	within regions	182	0.81532 Vb	96.66
	Total	184	0.84352	
**All countries**	Among countries	5	0.14091 Va	8.88
	Populations within countries	8	0.27001 Vb	17.01
	Within populations	692	1.17663 Vc	74.12
	Total	705	1.58755	

### Population history and demographic dynamics

The demographic pattern for each population and each country was examined. The Mean Absolute Error (MAE) values were moderate to low (Table 4). Results show negative non-significant Tajima’s D values for many populations. The exceptions are Misan, Mascara, Tiaret, Tlemcen, North-West Algeria, South Iraq region, Libya and the East, Central and West regions of Saudi Arabia, where we observed negative and significant Tajima’s D values. A negative Tajima’s D signifies an excess of low-frequency polymorphisms relative to expectation, indicating population size expansion (following a bottleneck or a selective sweep) or purifying selection. Fu’s *Fs*, also an index of population expansion, is known to be a more powerful tool than Tajima’s D [40]. Its power comes from the ability to differentiate between population growth and genetic hitchhiking, and in rejecting the hypothesis of neutral mutations. No significance values were found for the Harpending raggedness index (r) except for the Algerian samples, when they were grouped together. These results of MAE, Tajima’s D, Fu’s *Fs* and raggedness index (r) suggest a complex demographic history for our populations. We also performed mismatch distribution and Bayesian Skyline plot (BSP) analyses to gather more information regarding possible past population expansion.

**Table 4:** Neutrality and demographic expansion parameters

Population/Country	N	S	MAE	Tajima’s D (***P***-value)	Fu’s ***Fs*** (***P***-value)	Harpending r(***P***-value)
**Iraqi populations** **North-East**						
Sulimania [1]	9	0	0	0 (1.000)	0 (0)	0 (0)
**Central**						
Baghdad	51	18	0.564	−0.653 (0.280)	−1.380 (0.290)	0.123 (0.774)
Karbala	12	5	0.439	0.0918 (0.567)	0.538 (0.616)	0.155 (0.423)
Central [2]	63	20	0.440	− 0.911 (0.208)	−2.541 (0.166)	0.067 (0.469)
**South**						
Basra	11	1	0.055	−1.128 (0.137)	−0.410 (0.350)	0.438 (0.942)
Misan	24	11	0.502	−1.824 (0.016)	−0.090 (0.469)	0.203 (0.632)
South [3]	35	12	0.440	− 2.074 (0.001)	− 1.444 (0.161)	0.252 (0.688)
**Total**	107	22	0.432	−1.090 (0.134)	−5.070 (0.050)	0.055 (0.328)
**Algerian populations** **North-West**						
Mascara	20	3	0.270	−1.440 (0.032)	−2.135 (0.009)	0.187 (0.237)
Oran	17	5	0.560	−1.301 (0.077)	−2.953 (0.001)	0.211 (0.536)
Tiaret	11	2	0.391	−1.429 (0.037)	0.506 (0.622)	0.735 (0.933)
Tlemcen	18	15	0.557	−1.842 (0.015)	−2.089 (0.088)	0.053 (0.128)
North-West [1]	66	19	0.308	−2.255 (0.000)	−7.246 (0.000)	0.066 (0.096)
**Central**						
Adrar [2]	22	5	0.547	0.0671 (0.586)	0.928 (0.742)	0.280 (0.798)
**Total**	88	20	0.287	−2.092 (0.001)	−6.980 (0.004)	0.045 (0.048)
**Ethiopian populations** **North**						
Meseret	10	11	0.609	−1.202 (0.139)	−0.944 (0.237)	0.064 (0.138)
Mihquan	10	9	0.838	−1.411 (0.080)	2.172 (0.898)	0.226 (0.701)
North [1]	20	14	0.503	−1.390 (0.088)	−0.596 (0.419)	0.067 (0.269)
**Central-East**						
Adane	10	11	0.757	−0.741 (0.272)	0.505 (0.600)	0.176 (0.655)
Arabo	10	4	0.786	−0.339 (0.414)	−1.629 (0.051)	0.185 (0.462)
Horro	30	10	0.782	0.219 (0.638)	2.383 (0.867)	0.125 (0.647)
Jarso	14	0	0	0 (0)	0 (0)	0 (0)
Midir	10	2	0.586	−0.183 (0.322)	−0.272 (0.360)	0.240 (0.509)
Negasi Amba	10	1	0.297	0.819 (0.853)	0.818 (0.672)	0.222 (0.340)
Central-East [2]	84	20	0.781	−0.239 (0.497)	1.547 (0.786)	0.103 (0.748)
**West**						
Amshi	10	1	0.371	1.302 (0.919)	1.029 (0.789)	0.288 (0.612)
Ashuda	10	1	0.186	0.014 (0.754)	0.417 (0.670)	0.209 (0.326)
Batambie	8	1	0.100	−1.054 (0.187)	−0.182 (0.446)	0.312 (0.792)
Dikuli	10	2	0.586	−0.183 (0.355)	−0.272 (0.374)	0.240 (0.531)
Gafera	10	1	0.297	0.819 (0.827)	0.818 (0.698)	0.222 (0.366)
Surta	9	1	0.080	−1.088 (0.157)	−0.263 (0.389)	0.358 (0.918)
Tzion Teguaz	10	3	0.342	−0.431 (0.331)	0.345 (0.571)	0.133 (0.180)
West [3]	67	5	0.578	−0.104 (0.497)	−2.569 (0.092)	0.124 (0.290)
**South**						
Girissa	10	4	0.487	−0.942 (0.204)	−2.096 (0.019)	0.073 (0.081)
Kumato	10	10	0.819	−0.364 (0.361)	0.505 (0.583)	0.144 (0.527)
Loya	10	8	0.627	1.076 (0.865)	0.706 (0.646)	0.082 (0.250)
Shubi Gemo	10	14	0.721	0.746 (0.798)	0.723 (0.616)	0.159 (0.757)
South [4]	40	23	0.653	0.345 (0.703)	−4.181 (0.083)	0.020 (0.118)
**Total**	211	33	0.573	−0.938 (0.176)	−15.722 (0.001)	0.045 (0.364)
**Saudi Arabia**						
East [1]	45	18	0.729	− 1.964 (0.006)	−9.069 (0.000)	0.132 (0.494)
Central [2]	43	20	0.231	−2.207 (0.001)	− 4.717 (0.014)	0.048 (0.096)
West [3]	97	23	0.314	−1.862 (0.009)	−7.33 (0.007)	0.062 (0.200)
**Total**	185	34	0.375	−2.115 (0.000)	−18.713 (0.000)	0.061 (0.186)
**Other populations**						
**Libya**	23	14	0.520	−1.539 (0.047)	−3.704 (0.018)	0.058 (0.183)
**Pakistan**	92	24	0.547	−0.861 (0.210)	−4.096 (0.085)	0.063 (0.482)
**Total**						
**All the samples included in this study**	706	55	0.323	−1.668 (0.013)	−97.654 (0.000)	0.025 (0.096)

**Fig. 3:**
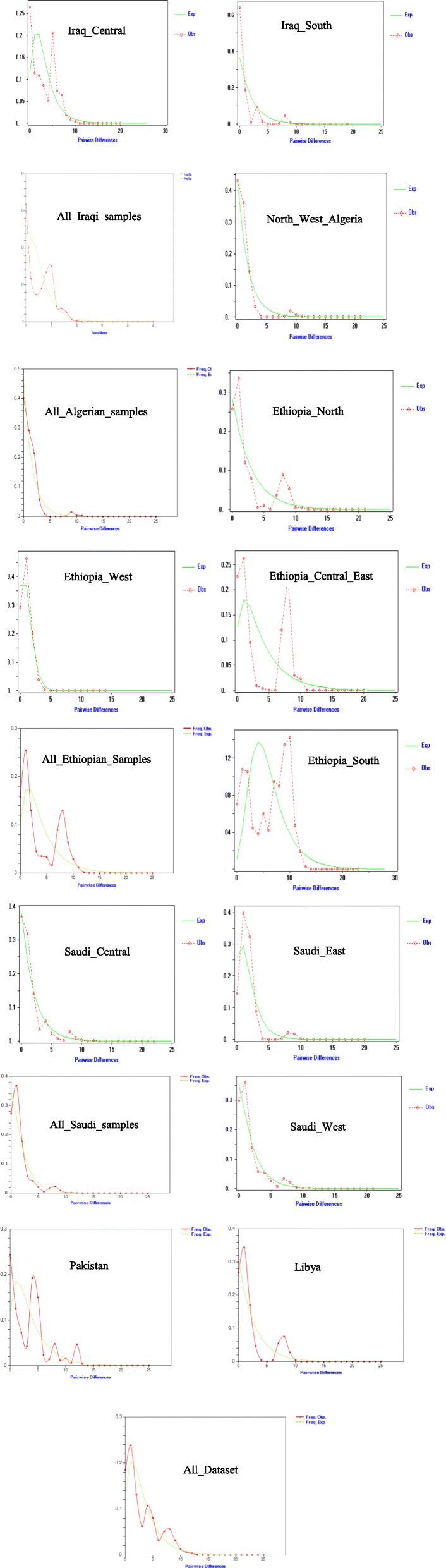
Mismatch distribution patterns for regions and countries

The mismatch distribution graphs show three patterns: uni-, bi- and multi-modal (Fig. 3 and Additional file 1: Fig. S10). The dominant distribution pattern is unimodal, and the multimodal the is least frequent one among the populations. These results are compatible with the demographic expansion results from Table 4, which suggest demographic expansion, bottleneck or purifying selection.

**Fig. 4:**
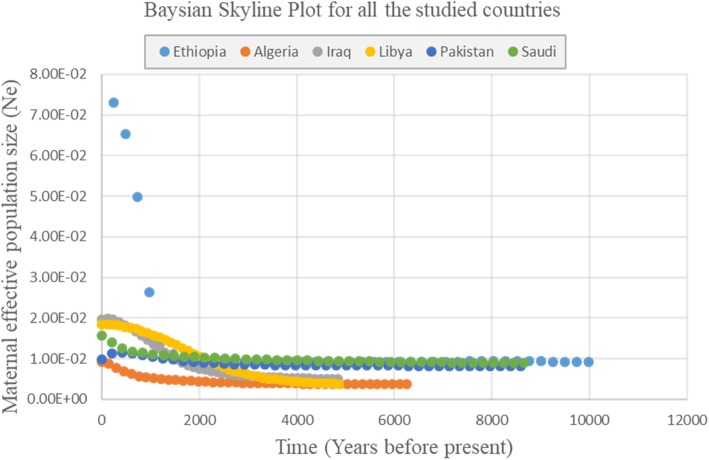
Bayesian Skyline Plot (BSP) for the countries. Points for each country represent the estimated effective population size at different time point

Bayesian Skyline Plots show evidence of demographic expansion for all the countries in recent times, except for Pakistan (Fig. 4). Ethiopia shows evidence of a rapid population increase in recent years, much higher than the ones observed for Iraq, Libya, Saudi Arabia and Algeria.

## Discussion

In this study, we analysed the mtDNA genetic diversity of 706 village chickens from Algeria, Ethiopia, Iraq, Libya, Pakistan and Saudi Arabia with the aim of unravelling their history and origin. To the best of our knowledge, it represents the first study on the *D*-loop mtDNA diversity of indigenous chicken from Algeria, Iraq and Libya. For Ethiopia, Pakistan and Saudi Arabia information about different populations (not included here) have been reported in another studies [15, 41, 42].

The history of chicken domestication remains unsettled. The Indian subcontinent as well as South-East Asia have been proposed as major centres of origins of domestic chicken, with for the former the Indus Valley a possible centre of domestication. Accordingly, we would expect that Pakistani chicken would show the highest *D*-loop mtDNA diversity. This is not the case when we consider all haplogroups together for each country (Table 1). It is also not the case when examining only haplogroup E (Additional file 1: Table S6), believes to have its centre of diversity on the Indian subcontinent and with Liu et al. [14] reporting it as being the commonest one in Europe and India. Considering all haplogroups or only haplogroup E, Ethiopia displays the largest diversity. This may be a consequence of the number of chickens examined here, with more than twice as many Ethiopian than Pakistani samples. Alternatively, or in addition, it may reflect multiple arrivals of chicken in East Africa following different dispersal routes.

Interestingly, previous studies have suggested a possible dual origin for the chicken of East Africa [15, 43], with a terrestrial origin from the Indian subcontinent but also a genetic influence from South-East Asian chickens following maritime trading routes across the Indian Ocean. The second most-frequent haplogroup is haplogroup D, postulated to be a legacy of the past Indian Ocean maritime trading networks [14]. This haplogroup is commonly observed in Ethiopian samples (Table 2), where it is more frequent in the southern region. It is completely absent in Libya and Algeria, and observed, but at a low frequency, in Iraq, Pakistan and Saudi Arabia. Such geographic distribution agrees with maritime routes of dispersal for this haplogroup across the Indian Ocean. Also, the absence of haplogroup D in Algeria and Libya, its relatively low frequency in other countries and its geographic distribution in Ethiopia is suggesting that the arrival and dispersal of the D haplogroup may have occurred after the North and Western dispersal of haplogroup E.

Within countries, we observed a different level of diversity across regions. Iraqi chicken populations from the Central area show more diversity than the two populations from the South of the country (Table 1). High genetic diversity in the Central region may be attributed to terrestrial inland route of dispersion and arrival of domestic chicken in the country rather than a maritime arrival in the South of the country. Also, it is worth remembering that the central area of the country includes the capital Baghdad, a city built during the eighth century as the capital of the Abbasid Caliphate. Accordingly, the region might have witnessed major movements of populations and their livestock from different geographic areas across time. Alternatively, it remains possible that domestic chicken may have reached today Iraq following a maritime trading route with subsequent admixture of populations more recently.

In Algeria, apart from one haplotype belonging to haplogroup A, only haplogroup E was observed. Diversity between the northern part of the country and the more central region is nearly the same. The populations studied here are geographically close to each other and in such context, the results might not be surprising. Examination of other populations from other parts of the country is needed to investigate whether Algerian chickens represent a single genetic group or not. In Libya, we only analysed a single population, and as for Algeria, only two haplogroups were observed, with haplogroup E being the commonest. Together, these results support a main Indian subcontinent origin for the chickens of these two countries and by extension to the shores of the Mediterranean Sea. It may have followed inland terrestrial routes throughout the Fertile Crescent or a more direct one through the Red Sea and Egypt.

Saudi Arabian populations show the presence of four haplogroups (A, C, D and E), with E the commonest. This probably reflects more than one route of introduction and origin for the Saudi Arabian chicken. Haplogroup E likely originated from the Indian subcontinent following a terrestrial and maritime route while other haplogroups may have reached the country following maritime trading network on the Arabian Gulf and the Red Sea. This is further supported by the genetic diversity values observed for the different regions, where the eastern part on the Arabian Gulf shows higher diversity (0.856 ± 0.044) compared to the central (0.631 ± 0.084) and western (0.702 ± 0.041) regions of the country.

Five haplogroups (A, B, C, D and E) have been identified in Ethiopian populations, with possibly different historical backgrounds of introduction. As mentioned before, the E haplogroup, the commonest, probably originated from the Indian subcontinent via either a terrestrial route along the Nile River Basin and/or arrival through the coastal areas of the Horn of Africa. The latter was the most likely route for the second most frequent haplogroup D. Within the country, the South region displays the highest haplotype (0.929 ± 0.021) and nucleotide (0.0150 ± 0.0006) variations compared to the other three Ethiopian regions. Then the Central - East, North and West regions with little differences among them.

Compared to other studies, the *D*-loop mtDNA diversity of Ethiopian, Iraqi, Libyan, Pakistani and Saudi chickens are higher than those previously reported for Iranian, Turkey and Egyptian chickens in the literature [44–46]. Similarly, among sub-Saharan chicken populations studied previously, we observe higher genetic diversity for the countries included in this study compared with populations from Nigeria, Chad, Uganda and Sudan [15, 22, 47]. These results need to be interpreted with caution considering difference in number of samples examined here. Nevertheless, for these other countries haplogroup E remains by far the commonest, with other haplogroups either rare or absent supporting our previous conclusion of arrival of haplogroup E on the African continent before other haplogroups. The high mtDNA diversity of Ethiopian chickens not only reflects extensive ancient livestock movements following trading routes linking Ethiopia to the Fertile Crescent civilisations, it also highlights the importance of the Horn of Africa as an entry point of livestock into the continent.

A high frequency of one haplotype (H_3) was found in all countries examined here (Fig. 2), which likely represent an ancestral E haplotype. Most samples were clustered in haplogroup E, while haplogroup A, B, C and D were observed at low frequencies. Haplogroup E and A were found in all the studied countries. Haplogroup B was found only in Ethiopia, represented by just one individual in the Mihquan population. Haplogroup C is present in Saudi Arabian and Ethiopian samples only, and specifically in the south-western region on the shores of the Red Sea (Abhaa and Jazan) in Saudi Arabia and in the south region (Loya) of Ethiopia. Haplogroups A and B have been reported before mainly from South China [14]. Haplogroup C was originally observed in chickens from Japan and South-East China dispersing through the maritime ancient trading network [14]. The origins of these haplogroups present a low frequency in our dataset remains speculative; they may be a legacy of ancient dispersal and/or more recent crossbreeding with commercial birds.

Overall, AMOVA showed similarity among countries, with most of the variation observed among individuals within populations (Table 3). However, more variation among populations within groups are observed for Ethiopia and Iraq in comparison to other countries. This supports our interpretation of haplotypes and haplogroups diversity discussed above, suggesting in particular for Ethiopia that multiple routes of chicken dispersal have occurred. It also indicates that once domestic chicken reached a country, genetic exchanges occurred within the country, limiting the usefulness of the *D*-loop mitochondrial DNA as a genetic marker for phylogeographic analysis within country.

## Conclusion

This paper presents for the first time the genetic diversity of Algerian, Iraqi and Libyan indigenous chickens using mtDNA *D*-loop sequencing information. Combined with findings from other studies, the results presented here add further support for a main Indian subcontinent origin for chickens of the countries examined here, as well as for the importance of the Indian Ocean maritime trading network for the dispersal of the species. However, no or low phylogeographic structure was observed overall across the studied populations, showing the limitation of the *D*-loop chicken mitochondrial DNA diversity for this purpose.

## Methods

### Collection of samples and genomic DNA isolation

**Fig. 5:**
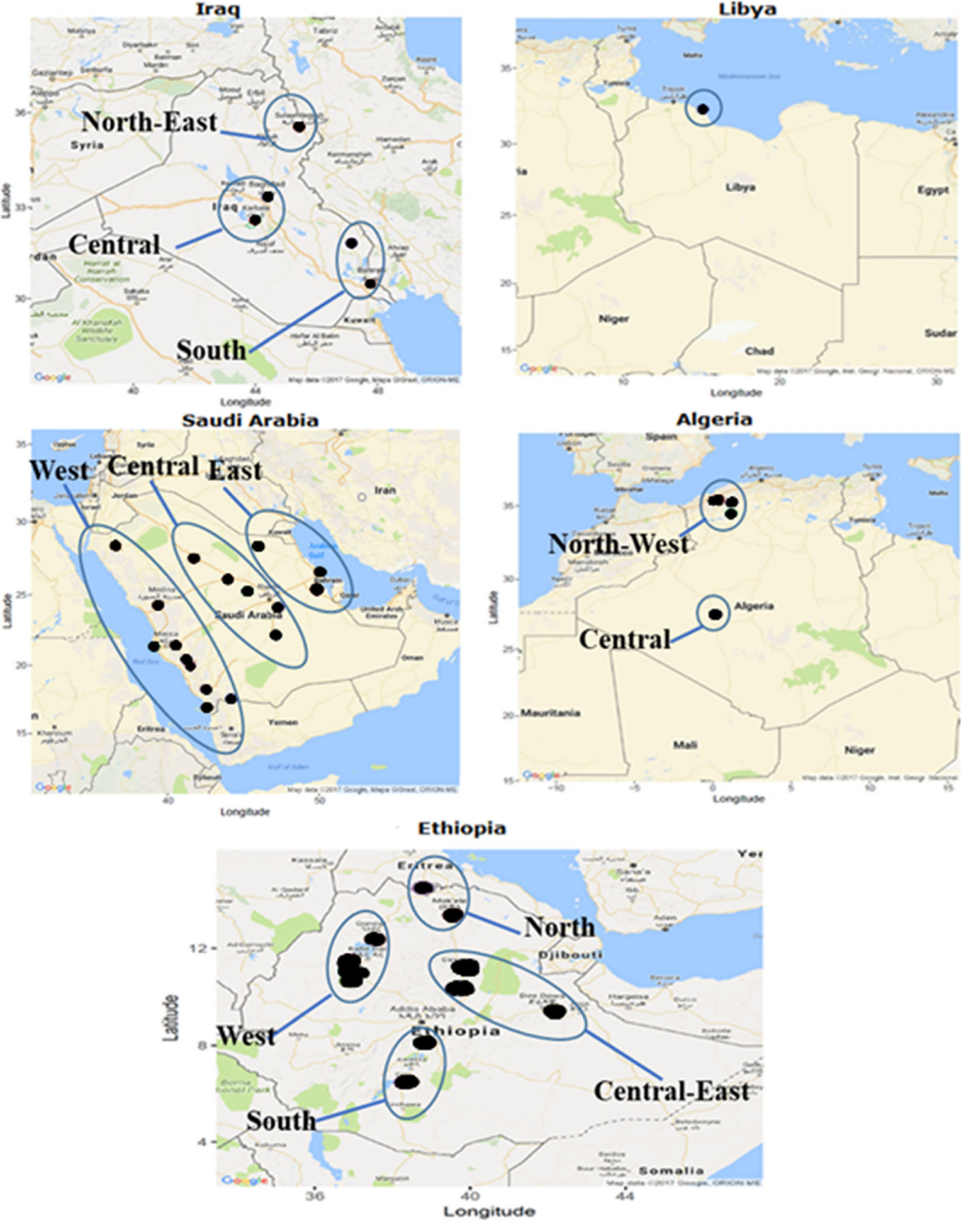
Sampling locations and grouping in regions for the countries included in this study

This study was conducted on 706 samples. It includes chicken from Pakistan (*n* = 92), Iraq (*n* = 107), Libya (*n* = 23), Saudi Arabia (*n* = 185), Algeria (*n* = 88) and Ethiopia (*n* = 211). Iraqi samples were collected from five different areas (Fig. 5) divided into three groups: (i) North, represented by Sulimania (n = 9) in the North-East part of Iraq; (ii) Central with two sampling locations in the central region of Iraq, Baghdad (*n* = 51) and Karbala (*n* = 12); and (iii) South with two regions in the South-East and southern part of the country, Misan (*n* = 24) and Basra (*n* = 11). In Saudi Arabia, samples were collected from 17 sites divided into three groups, East (Al-Qatef, Hafar Al-Batin and Al-Hessa (*n* = 45)), Central (Hail, Al-Aflaj, Al-Kharj, Al-Amariah and Unayzah (*n* = 43)) and West (Abha, Al-Baha, Jeddah, Jazan, Mecca, Medina, Najran, Tabuk and Taif, (*n* = 97)). Algerian chicken samples were divided into two groups, North (Mascara (*n* = 20), Oran (*n* = 17), Tiaret (n = 11) and Tlemcen (n = 18)) and Central (Adrar (*n* = 22)). Due to the political situation, sampling in Libya was limited to one population representing the North-West part of the country along the Mediterranean Sea.

In Ethiopia, chicken samples were collected from 19 different sites from both the highland and lowland parts of the country. Ten samples from each site were examined except for four populations (Batambie n = 8, Horro *n* = 30, Jarso *n* = 14 and Surta n = 9). The Ethiopian populations were divided into four groups: North (Meseret and Mihquan), Central-East (Adane, Arabo, Horro, Jarso, Midir and Negasi Amba), West (Ashuda, Amshi, Batambie, Dikuli, Gafera, Surta and Tzion Teguaz) and South (Kumato, Loya, Shubi Gemo and Girissa). All the blood samples were collected from free-range scavenging or semi-scavenging village chickens following standard veterinary practice approved by the relevant authority in each country and written or verbal consent from the farmers to sample their birds.

### DNA extraction, PCR amplification and sequence alignment

Total genomic DNA was extracted from air-dried blood preserved on FTA classic cards (FTA® cards) (Whatman Biosciences) using the MACHEREY-NAGEL DNA extraction kit or from full blood preserved in ethanol using the Qiagen kit following the manufacturers’ instructions.

Five hundred and forty-nine base pairs of the mtDNA *D*-loop region were amplified using AV1F2 (5′-AGGACTACGGCTTGAAAAGC-3′) [15] as the forward primer, and H547 (5′- ATGTGCCTGACCGAGGAACCAG-3′) [16] as the reverse primer. PCR amplifications were carried out in a 20-μl reaction volume containing 40 ng genomic DNA, 10 μl PCR ready master mix (Thermo Scientific Ltd), 0.5 μM of each primer, and sterile nuclease-free water to reach the final volume of 20 μl. The PCR was carried out using a Peltier thermocycler with the following conditions: Hot lid (110 °C), hot start 98 °C (30 s), denaturation 98 °C (5 s), annealing 63 °C (5 s), elongation 72 °C (10 s), 35 cycles and final extension step at 72 °C (1 min) [17–19]. The PCR product was electrophoresed on a 1% agarose gel at 100 V for 45 min, and then the gel was stained by 1% ethidium bromide and visualised under ultraviolet light. Amplified DNA fragment size was estimated through size comparison with a 1 kb DNA ladder from New England BioLabs loaded alongside the PCR products. The products were purified using the reSource PCR purification kit from Source Bioscience.

An Applied Biosystems 3730 DNA Analyser was used for Sanger sequencing. For each sample, the primer sequences were trimmed to generate the 549-bp sequence fragment and correct possible base-calling errors using the proseq3 version 3.5 software [20]. Sequences were aligned to the chicken mtDNA reference sequence (GenBank accession no. AB098668) [16] using Clustal X version 2.1 [21]. Analyses were restricted to the first 397 bp of the sequence, which includes the hypervariable region (HV1) of the *D*-loop [22]. This dataset for the six countries was deposit in GenBank sequence database (https://www.ncbi.nlm.nih.gov/genbank/), accession numbers MK920994-MK921699.

For all the Ethiopian and some of the Iraqi samples (*n* = 22), the full mtDNA was retrieved from full-genome sequencing data using the Genome Analysis Toolkit (GATK V3.7) [23, 24]. The sequences were then aligned with the chicken mtDNA reference genome sequence and the 397 bp of the *D*-loop selected for further analysis.

### Genetic diversity estimation

DnaSP v5 was used to identify polymorphic sites, the number of haplotypes and to calculate haplotype diversity (Hd), nucleotide diversity (*π*) and the average number of nucleotide differences [25]. These parameters were examined both at population and country levels. The statistical significant differences for haplotype and nucleotide diversities were tested following Alexander et al. [26] methodology.

### Phylogenetic analysis

In order to assess the possible phylogeographic origin of the samples, reference sequences from Liu et al. [14] haplogroups were included (AB114069 – haplogroup A, AB007744 – haplogroup B, AB114070 – haplogroup C, AY588636 – haplogroup D, AB114076 – haplogroup E, AF512285 – haplogroup F, AF512288 – haplogroup G, D82904 – haplogroup H, and AB009434 – haplogroup I). The phylogenetic tree was constructed using jModeltest version 2.1.7 [27] to predict the best-fit model and Phyml version 3.0 for the maximum likelihood tree [28]. The confidence level for each branch in the tree was assessed with 1000 bootstrap replications. To infer the relationship between the haplotypes, Median-Joining (MJ) network was built using the NETWORK 5.0.0 [29] and the PopArt [30]. To further illustrate the ancient migratory routes of chicken, 775 sequences from Chad, Egypt, Nigeria, Sudan, Turkey and Iran were downloaded from GeneBank (Table S1).

### Analysis of molecular variance (AMOVA)

The analysis of molecular variance was implemented using Arlequin v 3.5.2 with 1000 permutations [31]. It was performed across countries and within countries. Across countries, the analysis was performed using all the samples in a country with each country as an individual group. For within-country analyses, we followed the grouping of the populations as described in the sampling section, namely Iraqi samples were divided into three groups (Central *n* = 63, North *n* = 9, South *n* = 35), Algerian samples were divided into two groups, (North-West and Central). Ethiopian samples were divided into four groups ((Meseret and Mihquan), (Adane, Arabo, Horro, Jarso, Midir and Negasi Amba), (Ashuda, Amshi, Batambie, Dikuli, Gafera, Surta, and Tzion Teguaz), and (Girissa, Kumato, Loya, Shubi Gemo)), and Saudi Arabian were divided into three groups (East (*n* = 45), Central (*n* = 43) and West (*n* = 97)). All Libyan and Pakistani samples were grouped as a single Libyan or Pakistani country population.

### Neutrality test and demographic dynamics

The demographic profiles for each population were calculated from mismatch distribution patterns [32]. The Mean Absolute Error (MAE) was calculated between the observed and the theoretical (expected) mismatch distributions to provide support for demographic expansion [33]. Then, Fu’s *Fs* [34] and Tajima’s D [35] were estimated using the infinite site model in DnaSP v.5 [25].

Bayesian Skyline Plots (BSPs) [36] were investigated in order to have deeper insight into the demographic history of the chicken within countries. It was accomplished using the piecewise constant function in BEAST V 2.4.7 [37]. First, the HKY + G nucleotide substitution model was used for the analysis and then separate Markov Chain Monte Carlo simulation (MCMC) runs were applied for twenty-million generations sampled every one-thousand generations with the first two-million generations used as burn-in. Tracer software V.1.7 [38] was used to calculate the convergence of the posterior estimates of *Ne* to the likelihood of stationary distribution. The BSPs were standardised using the molecular mutation rate of evolution for chicken mtDNA, 3.13 × 10^− 7^ mutations/site/year (m/s/y) following Alexander et al. [39]. This estimation of BSPs was performed on each country samples separately and they were then plotted together.

## Supplementary information

**Additional file 1: Fig. S1.** mtDNA *D*-loop variation of 88 haplotypes found in the 706 village chicken. **Fig. S2.** Median-Joining network for Algerian haplotypes (*n* = 13). **Fig. S3.** Median-Joining network for Ethiopian haplotypes (*n* = 36). **Fig. S4.** Median-Joining network for Iraqi haplotypes (*n* = 18). **Fig. S5.** Median-Joining network for Libyan haplotypes (*n* = 10). **Fig. S6.** Median-Joining network for Pakistani haplotypes (*n* = 19). **Fig. S7.** Median-Joining network for Saudi Arabia haplotypes (*n* = 26). **Fig. S8.** Maximum likelihood tree for the 136 haplotypes and references from Liu et al. [14]. **Fig. S9.** Median-Joining network for 136 haplotypes of the collected and downloaded samples. **Fig. S10.** Mismatch distribution patterns of populations included in this study. **Table S1.** Downloaded sequences from GeneBank. **Table S2a.** Haplotype diversity significant differences among Iraqi populations. **Table S2b.** Nucleotide diversity significant differences among Iraqi populations. **Table S3a.** Haplotype diversity significant differences among Algerian populations. **Table S3b.** Nucleotide diversity significant differences among Algerian populations. **Table S4a.** Haplotype diversity significant differences among Ethiopian populations. **Table S4b.** Nucleotide diversity significant differences among Ethiopian populations. **Table S5a.** Haplotype diversity significant differences among Saudi regions. **Table S5b.** Nucleotide diversity significant differences among Saudi regions. **Table S6.** Location, sample size and genetic diversity of haplogroup E.

## Data Availability

The datasets generated and/or analysed during the current study are available in the GenBank repository, [https://www.ncbi.nlm.nih.gov/genbank/] accession numbers MK920994–MK921699.

## Abbreviations

AMOVA: Analysis of Molecular Variances
BSP: Bayesian Skyline Plot
FTA Cards: Flinders Technology Associates Cards
GATK: Genome Analysis Toolkit
Hd: Haplotype diversity
MAE: Mean Absolute Error
MCMC: Markov Chain Monte Carlo
MJ: Median-Joining
PCR: Polymerase Chain Reaction
휋: Nucleotide diversity
