# Supplementary material for: The maternal origin of indigenous domestic chicken from the Middle East, the north and the horn of Africa
Source: BMC Genet. 2020 Mar 14;21:30. doi: 10.1186/s12863-020-0830-0 (PMC7071775; doi:10.1186/s12863-020-0830-0)
Supplement: Supplementary file 1 — Additional file 1: Fig. S1. mtDNA D-loop variation of 88 haplotypes found in the 706 village chicken. Fig. S2. Median-Joining network for Algerian haplotypes (n = 13). Fig. S3. Median-Joining network for Ethiopian haplotypes (n = 36). Fig. S4. Median-Joining network for Iraqi haplotypes (n = 18). Fig. S5. Median-Joining network for Libyan haplotypes (n = 10). Fig. S6. Median-Joining network for Pakistani haplotypes (n = 19). Fig. S7. Median-Joining network for Saudi Arabia haplotypes (n = 26). Fig. S8. Maximum likelihood tree for the 136 haplotypes and references from Liu et al. [14]. Fig. S9. Median-Joining network for 136 haplotypes of the collected and downloaded samples. Fig. S10. Mismatch distribution patterns of populations included in this study. Table S1. Downloaded sequences from GeneBank. Table S2a. Haplotype diversity significant differences among Iraqi populations. Table S2b. Nucleotide diversity significant differences among Iraqi populations. Table S3a. Haplotype diversity significant differences among Algerian populations. Table S3b. Nucleotide diversity significant differences among Algerian populations. Table S4a. Haplotype diversity significant differences among Ethiopian populations. Table S4b. Nucleotide diversity significant differences among Ethiopian populations. Table S5a. Haplotype diversity significant differences among Saudi regions. Table S5b. Nucleotide diversity significant differences among Saudi regions. Table S6. Location, sample size and genetic diversity of haplogroup E. [file 12863_2020_830_MOESM1_ESM.docx]

011111111122222222222222222222222222222333333333333333333333333

034677799901112222233333444444556689999000111112234555555666799

632717838970271245934689023679461511269268034572702234589237216 N

Ref TTCTTACCCTACATCATCCCCTGACGTTAATTCCAAATTCTCCCCTATTCACATTACCCTACT

H_1 ............G....T.........C.........C....T..C................. 12

H_2 .............C.G.C........C..G.CTT....................C........ 5

H_3 .............C...C........C....CT.............................. 291

H_4 .............C.G.C........C..G.CT.G...................C........ 43

H_5 .............C...C........C....CT.....................C.......C 3

H_6 ...........T..............C....CT.........................T.... 1

H_7 ...C.........C...C........C....CT........A..................... 1

H_8 ...C.........C...C....A...C....CT.............................. 1

H_9 ...C.........C...C........C....CT.............................. 10

H_10 .............C.G.C........C....CT................T............. 15

H_11 ...C......................................C.................... 7

H_12 ...C...................G..................C.................... 1

H_13 .............C...C........C....AT.............................. 6

H_14 .............C...C........C....CT................T............. 9

H_15 .............C...C........C....CT.................G............ 15

H_16 .............C...C........C.....T.............................. 13

H_17 .............C...C........CT...CT.............................. 33

H_18 ...C.............C........C....C..G.....C.........G......TT.... 7

H_20 .................C........C....C..G.....C.........G......TT.... 21

H_21 .............C...C........CT...CT...G.......................... 2

H_22 .............C...C........CT...CT..............C............... 6

H_23 .............C...C........C....CT.............................C 54

H_24 .......A.....C...C........C....CT.............................. 8

H_25 .............C...C........C.....T.............................C 1

H_26 ...C.......T..............................C.................... 8

H_27 .............C...C........................C.................... 9

H_28 .............C...C........C....CT......................G....... 1

H_29 .............C...C.T......C....CT.............................. 2

H_30 ...........T.C...C.T......C....CT.............................. 1

H_31 ...........T..............CT...CT.............................. 6

H_32 ...........T.C...C........C....CT.............................. 1

H_33 ............A....C.........T..............C..T................. 1

H_34 .........C...C...C........C.....T.............................. 3

H_35 ...C.........C...C........C....CT.......................T.....C 2

H_36 ...C.........C...C........C....CT.G.....C.........G......TT.... 1

H_37 ...C.........C...C........C....CT.........C...................C 1

H_38 .C...........C...C........C....CT.............................. 8

H_39 .................C.......AC....C..G...............G.......TC... 1

H_40 .............C...C........C....CT....................C......... 1

H_41 .............C...C........C....CT............................T. 3

H_42 ..........G..C...C........C....CT.............................. 2

H_43 .............C.G.C........C....CT.....................C........ 1

H_44 .............C...C........C....CT.....................C........ 1

H_45 ..T..............C........C....C..G.....C.........G......TT.... 2

H_46 ............A....C.......AC....C..G.......................TC... 2

H_47 ...C.............C........C....C..G.....C.........G......TTC... 1

H_48 .................C........C....CT.G.....C.........G......TT.... 3

H_51 .................C........C....CT.G.....C...................... 6

H_52 .................C........C.G..CT.G.....C...................... 1

H_53 ...C..T..........C........C....C..G.....C...T.....G......TT.... 2

H_55 .............C...C........C....CT...........................GT. 1

H_56 ...C.......T..............................C...........C........ 1

H_57 .............C...C.............CT.............................. 6

H_58 .................C........C....C..G.G...C.........G......TT.... 1

H_59 ..................................G.....C...................... 1

H_60 ...C.........C...C........C....C............................... 1

H_61 .............C...C........C....C............................... 2

H_62 .............C...C........C....C.......................G....... 1

H_63 .........C...C...C........C....CT.............................. 6

H_64 .............C...C........C....CT................T........T.... 1

H_65 ............A....C........C....CT.G.....C...................... 1

H_66 ...C.........C.G.C........C....CT................T............. 1

H_67 .............C.G.C..T.....C..G.CT..C..................C........ 1

H_68 .............C.G.C........C..G.CT.....................C........ 4

H_69 C............C.G.C........C..G.CT.....................C........ 3

H_70 ...........T..............C....CT..........T................... 1

H_71 .....G.....T..............CT...CT.............................. 1

H_72 .............C...C........C....CT...............C.............. 1

H_73 .............C...C........C....CT..........T......G............ 7

H_74 ........T....C...C........C....CT..........T......G............ 1

H_75 .............C...C........C....CT....T.....T......G............ 1

H_76 .............C...C........C....CT..........T................... 1

H_77 .............C...C........C....CT.......................T.....C 1

H_78 .............C...C........C....CT....T........................C 1

H_79 ...C.........C...C........C....CT.............................C 3

H_80 ...C.........C...C........C....CTT............................C 1

H_81 .............C.G.C........C..G.CT.G...............G...C........ 2

H_82 .............C.G.C........C....CT.G...................C........ 2

H_83 ...C.............C........................C.................... 1

H_84 .............C...C........C....CT.........C.................... 2

H_85 .............C.GCC........C..G.CT.G...................C........ 1

H_86 .............C...C........C....CT...................G.......... 5

H_87 ....C........C...C........C....CT.............................. 2

H_89 .............CT..C........C....CT.............................. 2

H_90 ............AC...C........C....CT.............................. 1

H_91 .....G.......C...C........C....CT.............................. 1

H_92 .............C...C.......AC....CT.............................. 1

H_93 ...........T..............C....CT.............................. 1

**Fig. S1**: The vertical numbers indicate to the position of the polymorphism compared to the mtDNA sequence of reference (GenBank accession no. AB098668). The right column under ‘N’ the number of times a haplotype was observed. Dots (.) indicate that the same nucleotide compared to the reference sequences was found, the letters show the polymorphism. The missing haplotypes number (19, 49, 50, 54 and 88 are for haplogroups D, F, G, I and H reference haplotypes (not added to the figure).


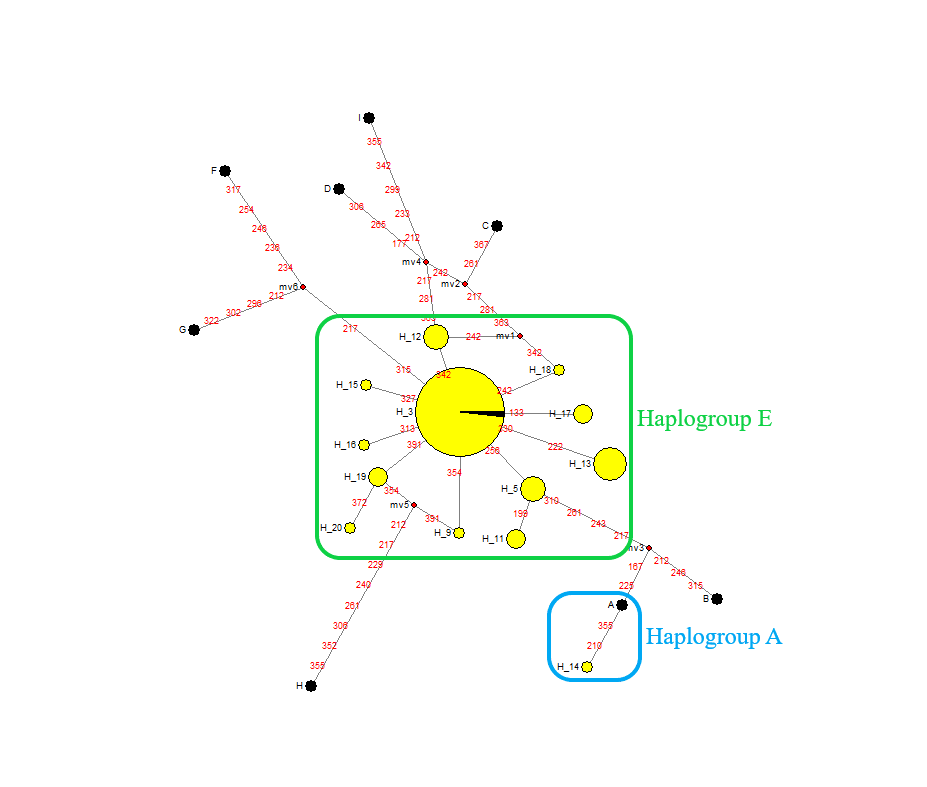


**Fig. S2:** The black circles refer to reference haplotypes, yellow = Algerian haplotypes. The numbers on the branch indicate the position of the mutations, the circles are proportional to the numbers of haplotypes.


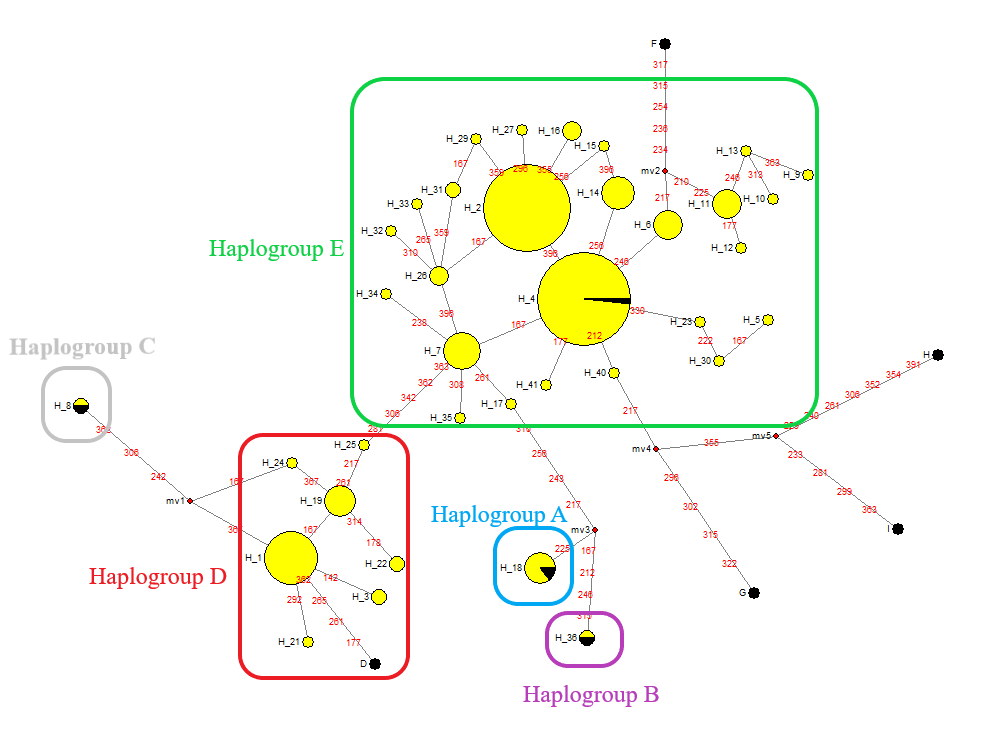


**Fig. S3:** The black circles refer to reference haplotypes, yellow = Ethiopian haplotypes. The numbers on the branch indicate the position of the mutations, the circles are proportional to the numbers of haplotypes.


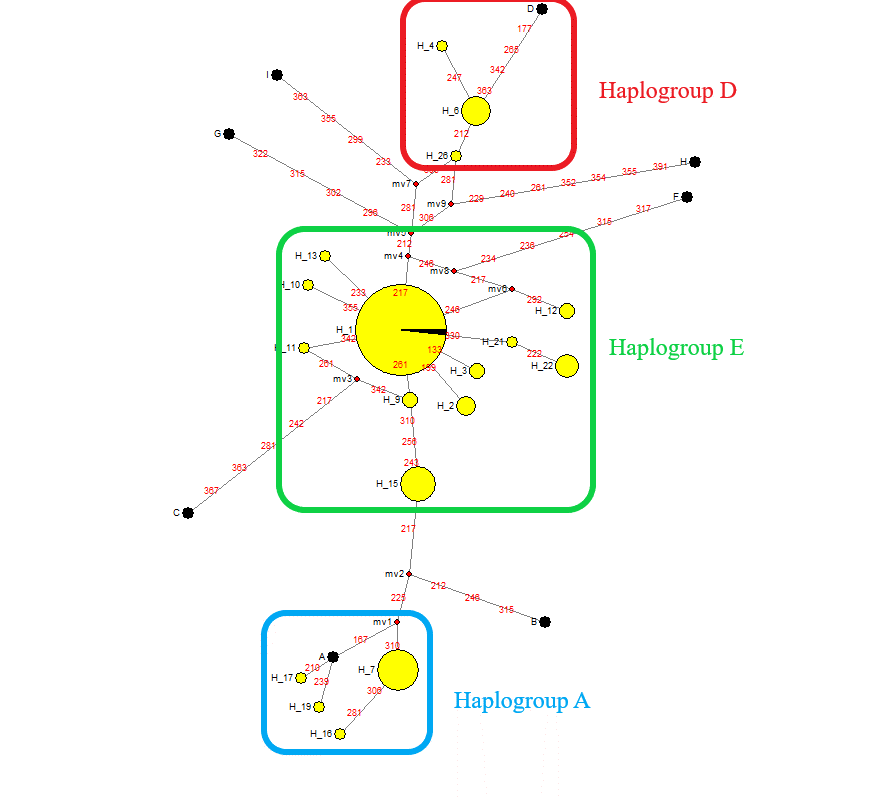


**Fig. S4:** The black circles refer to reference haplotypes, yellow = Iraqi haplotypes. The numbers on the branch indicate the position of the mutations, the circles are proportional to the numbers of haplotypes.


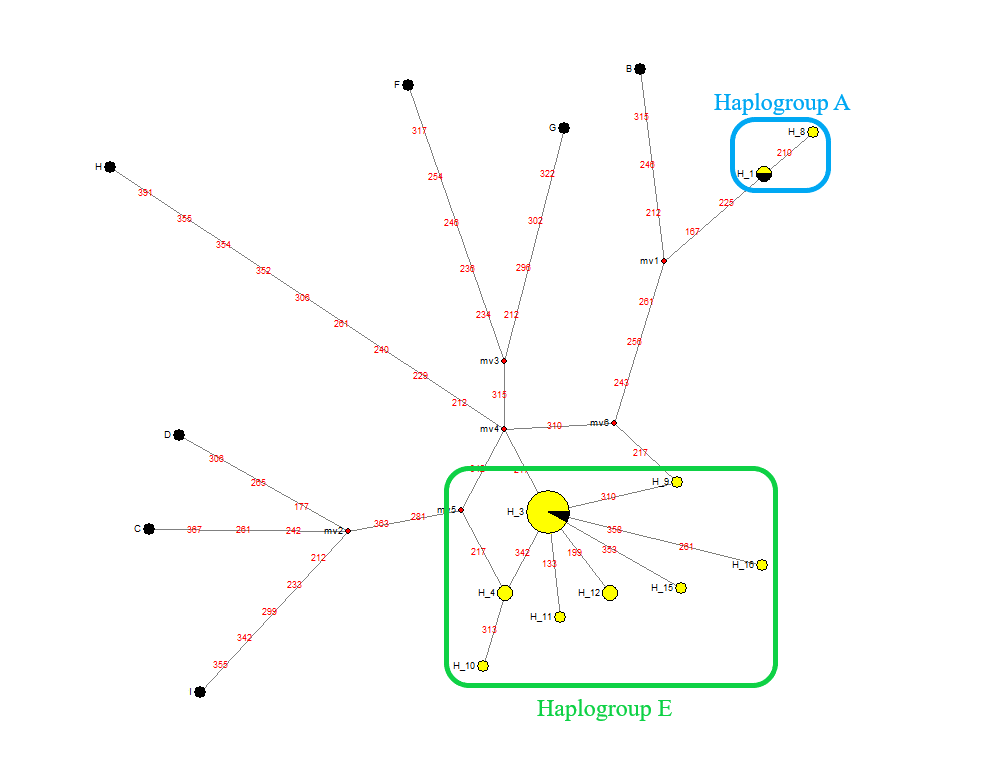


**Fig. S5:** The black circles refer to reference haplotypes, yellow = Libyan haplotypes. The numbers on the branch indicate the position of the mutations, the circles are proportional to the numbers of haplotypes.


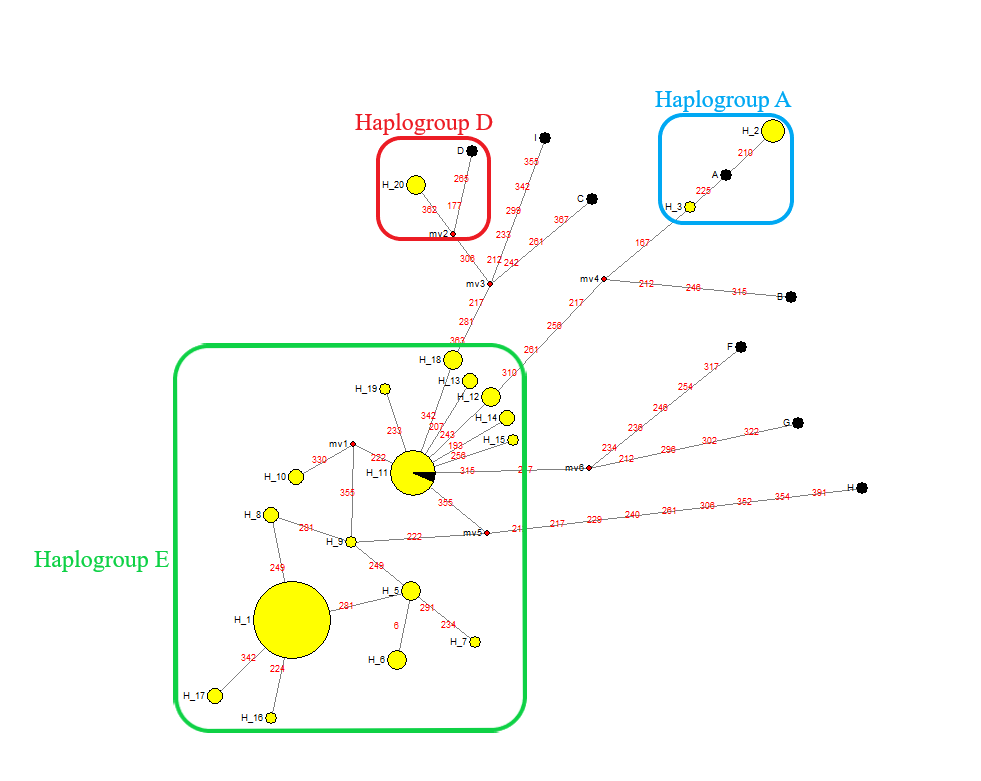


**Fig. S6:** The black circles refer to reference haplotypes, yellow = Pakistani haplotypes. The numbers on the branch indicate the position of the mutations, the circles are proportional to the numbers of haplotypes.


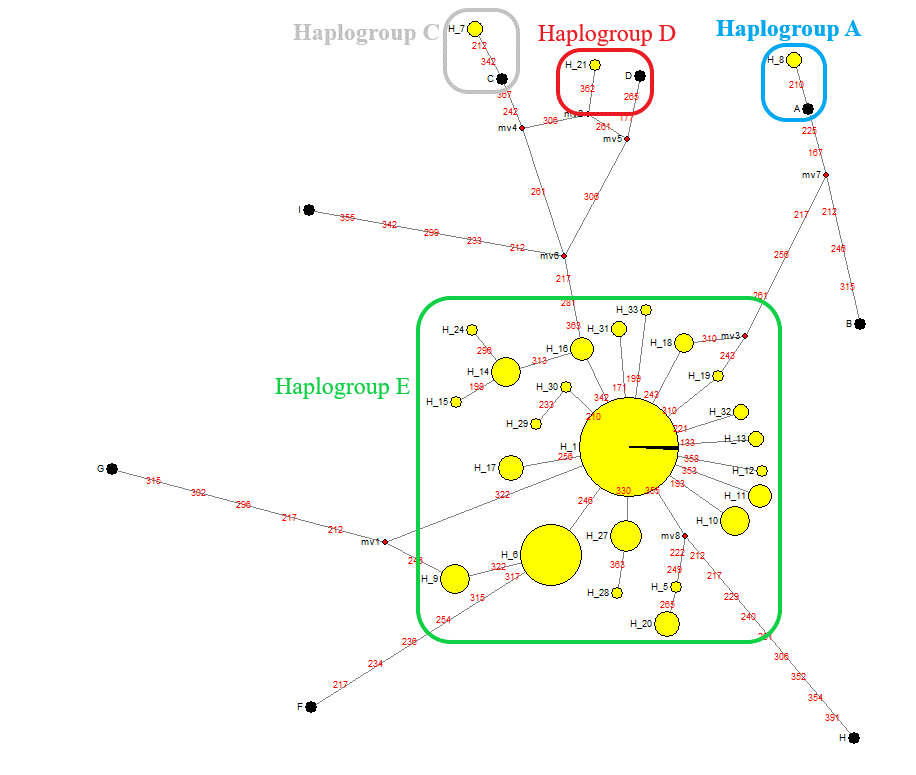


**Fig. S7:** The black circles refer to reference haplotypes, yellow = Saudi haplotypes. The numbers on the branch indicate the position of the mutations, the circles are proportional to the numbers of haplotypes.


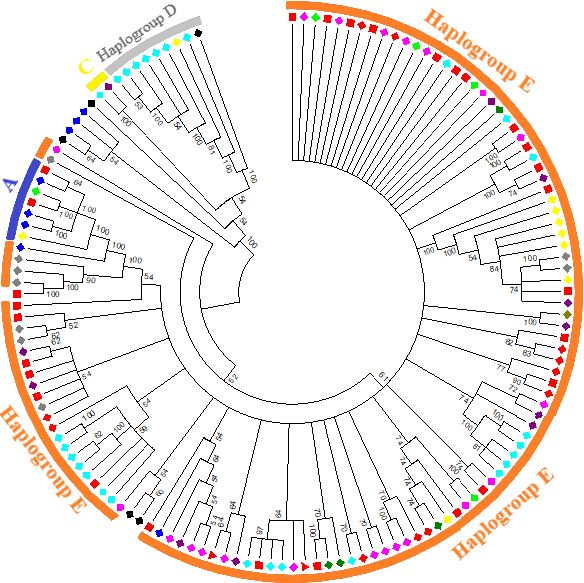


**D**

**B**

References

**Fig. S8:** = Iraqi haplotypes, = Ethiopian haplotypes, = Algerian haplotypes,

= Saudi haplotypes, = Pakistani haplotypes, = Libyan haplotypes, = common haplotypes among countries, = References, = Sudanese haplotypes. = Turkey & Iranian haplotypes, = Egyptian haplotypes, = Chadian haplotypes and = Nigerian haplotypes. Numbers on nodes represent bootstrap values.


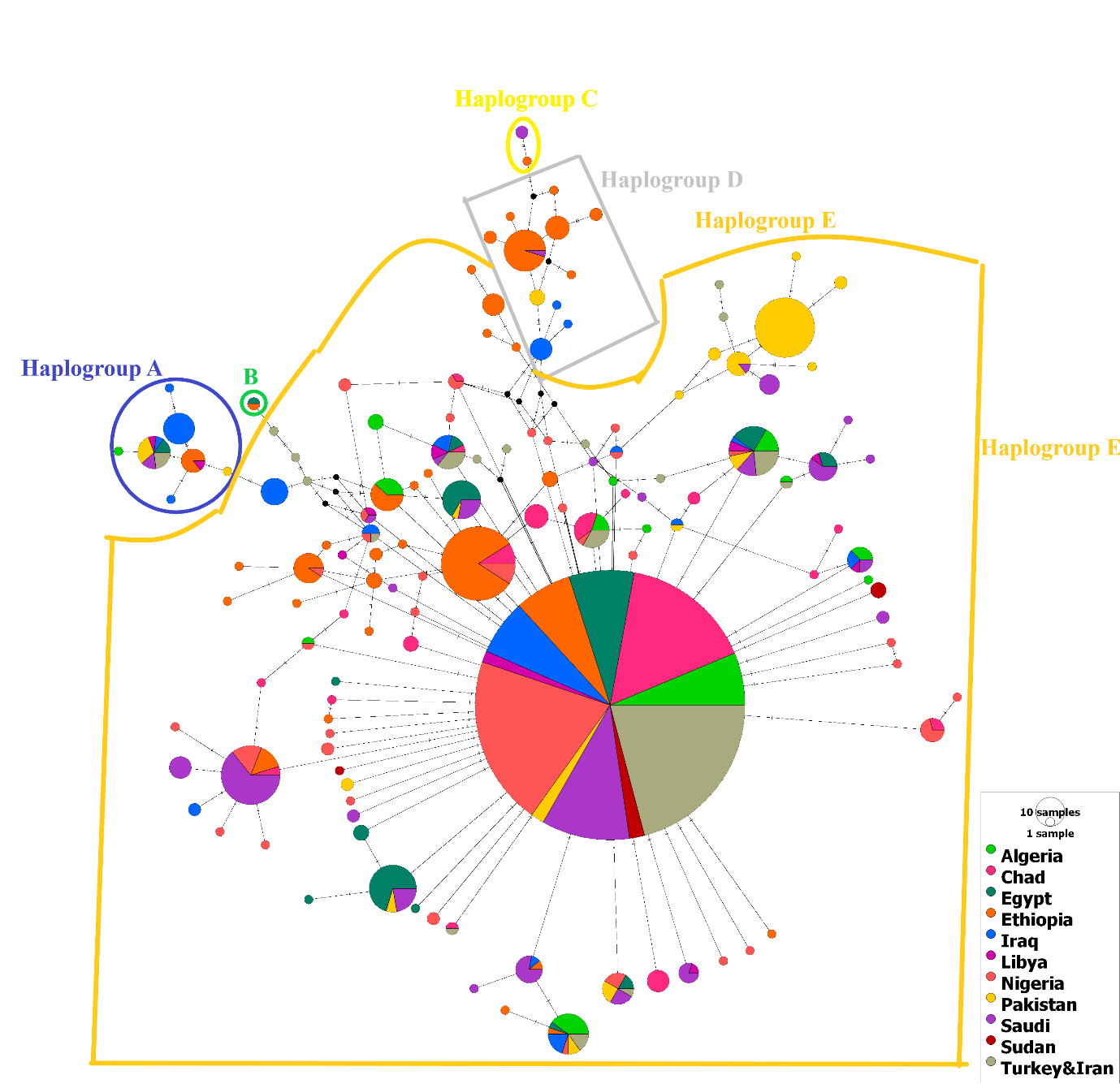


**Fig. S9**


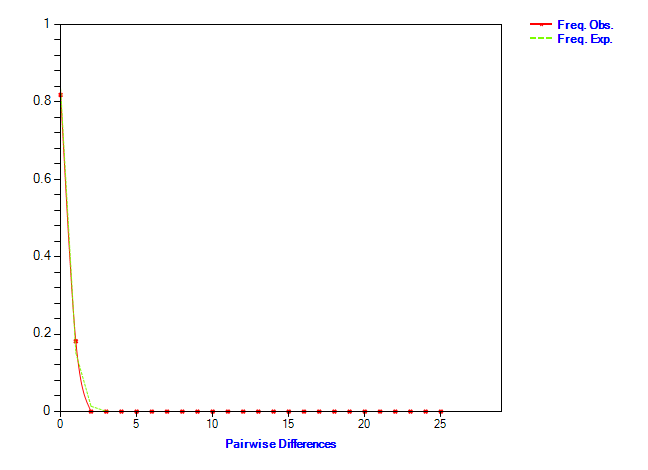

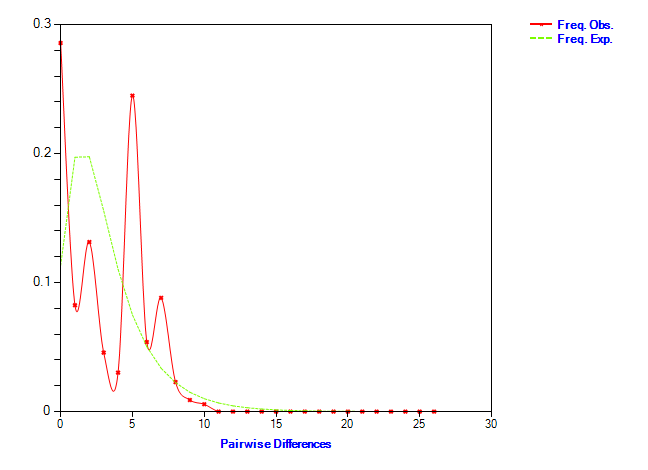


Basra

Baghdad


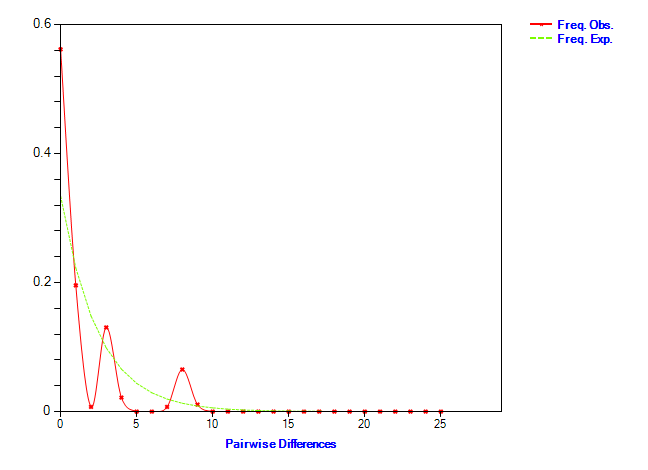

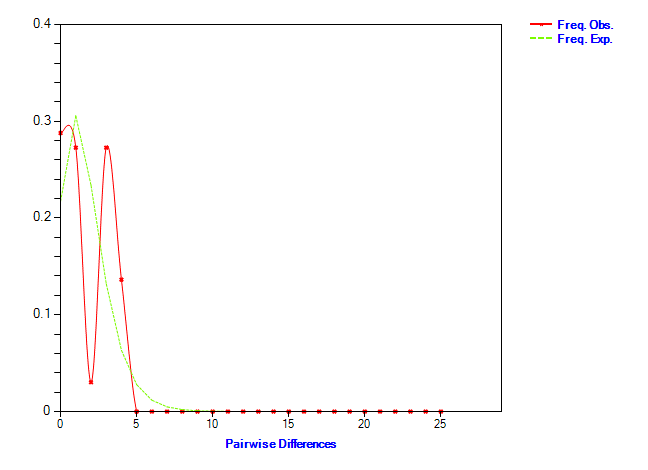


Misan

Karbala

**
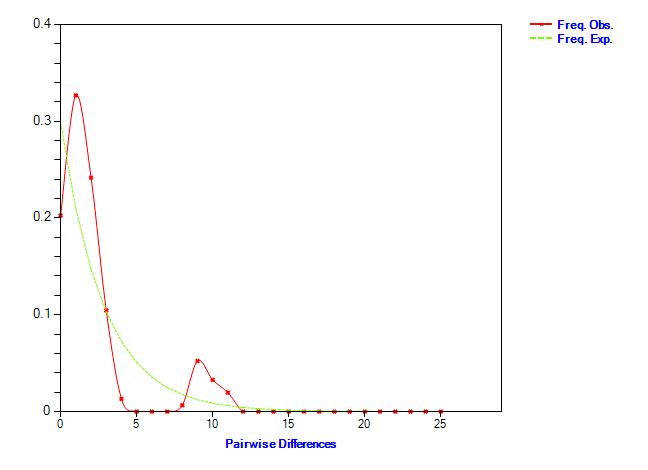
**

**
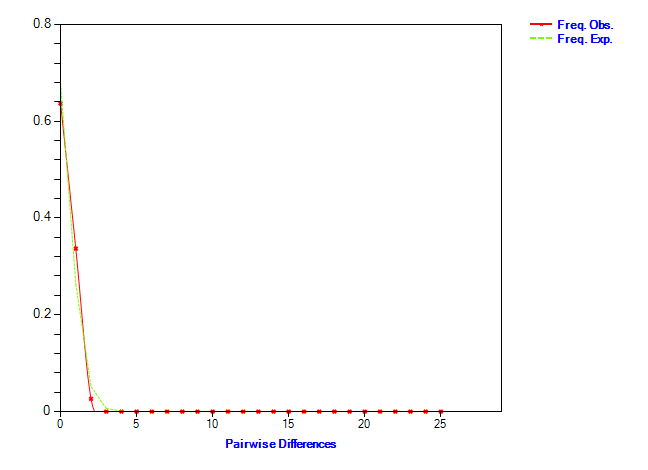
**

Mascara

Tlemcen


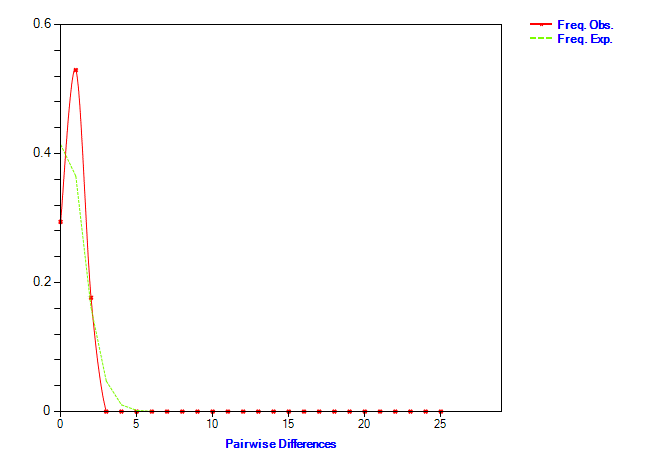

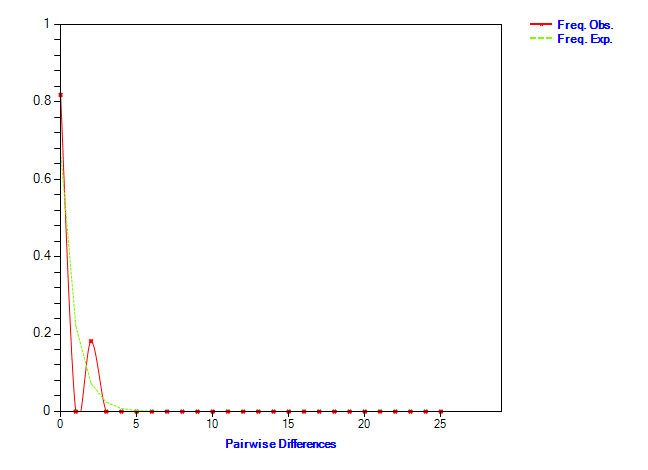


Oran

Tiaret


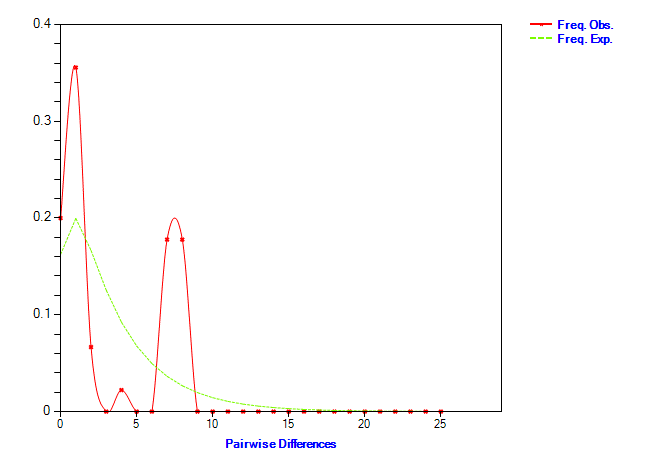
**
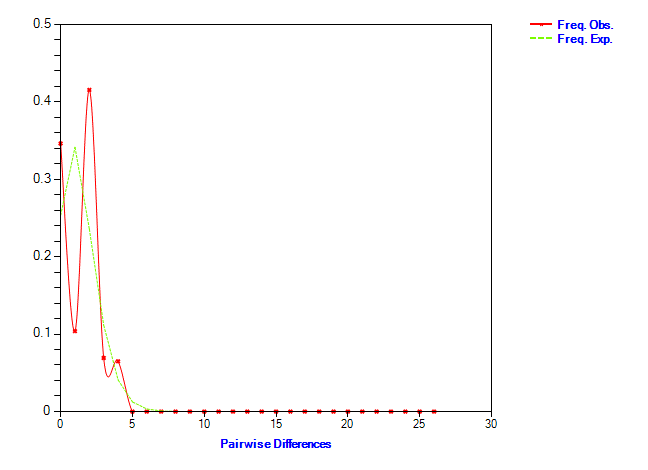
**

Adrar

Adane


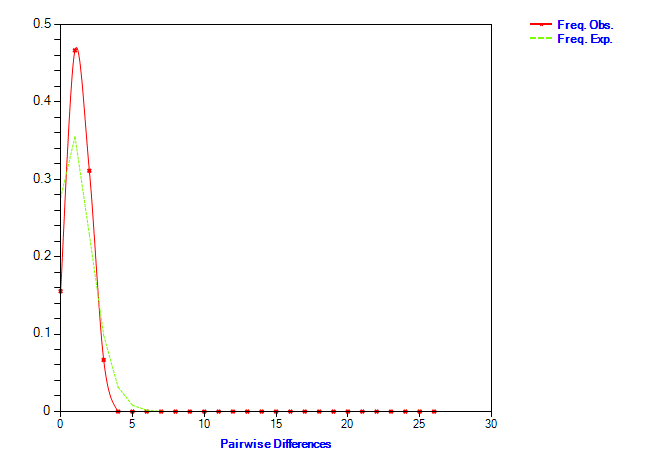

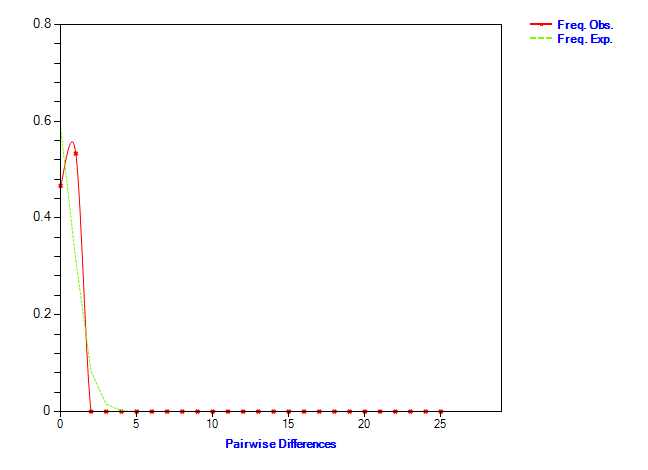


Amshi

Arabo


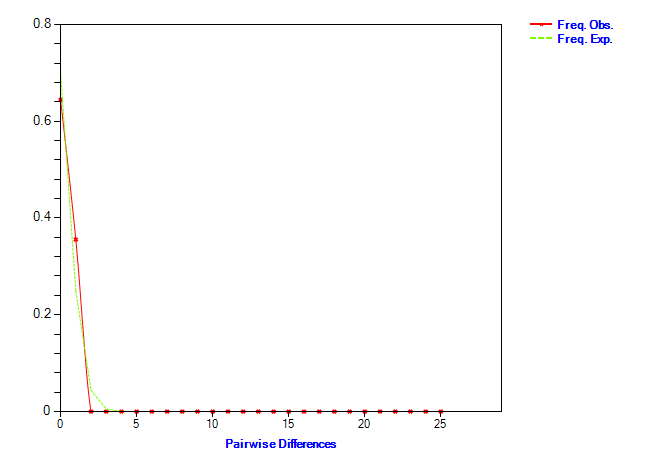

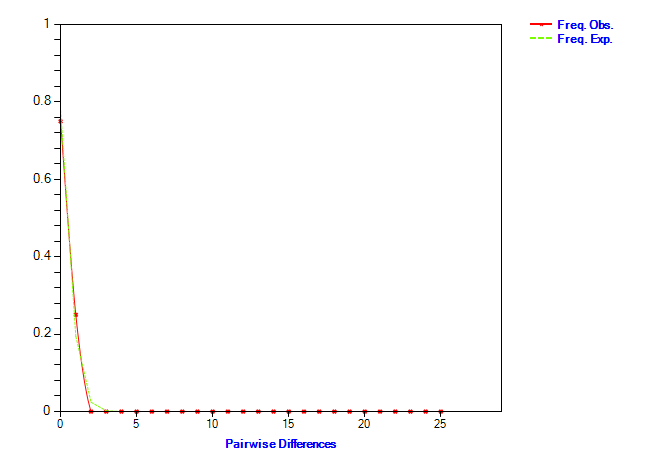


Ashuda

Batambie


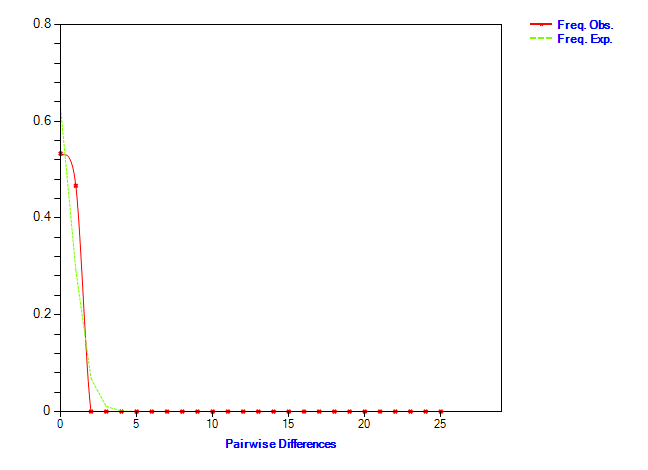

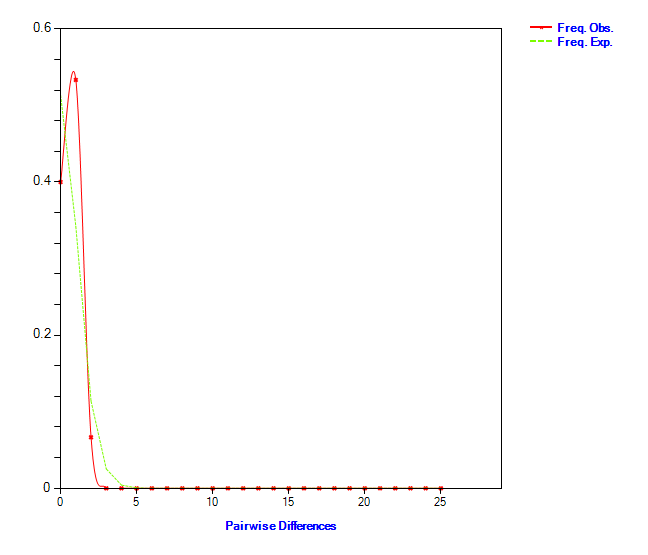


Gafera

Dikuli


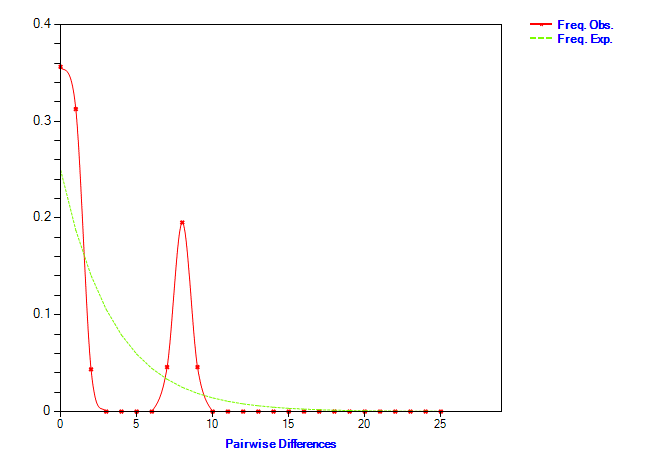

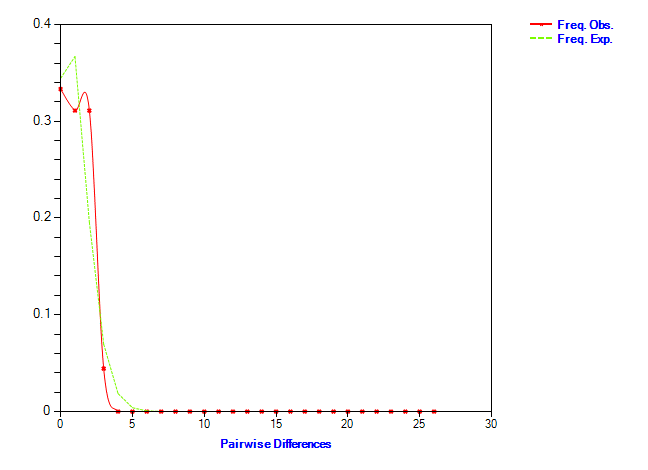


Horro

Girissa


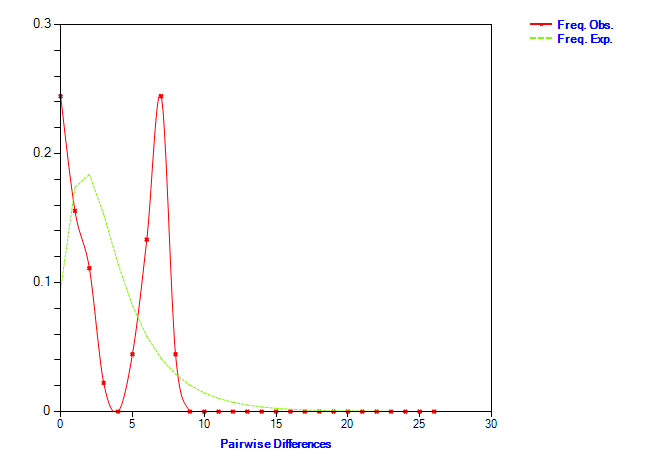


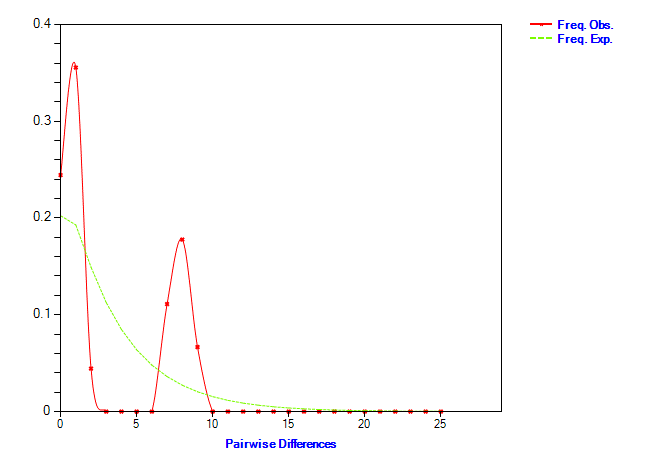


Loya

Kumato


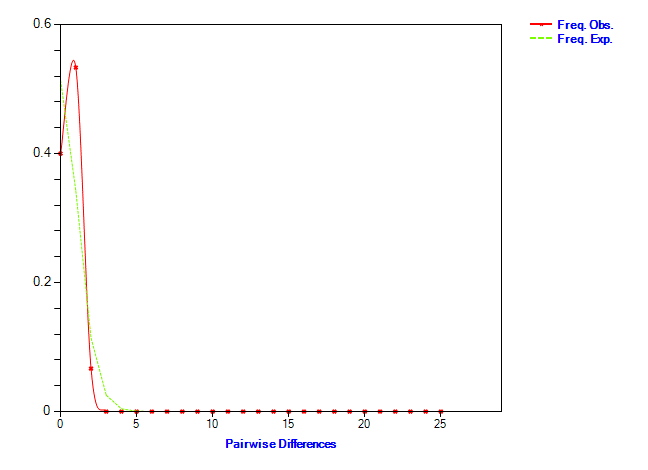

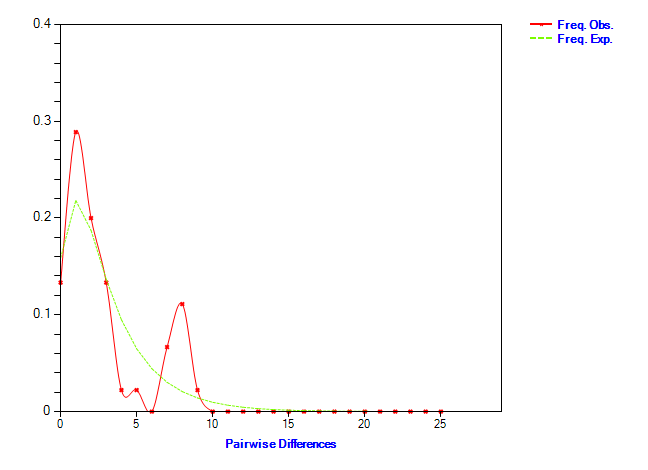


Meseret

Midir


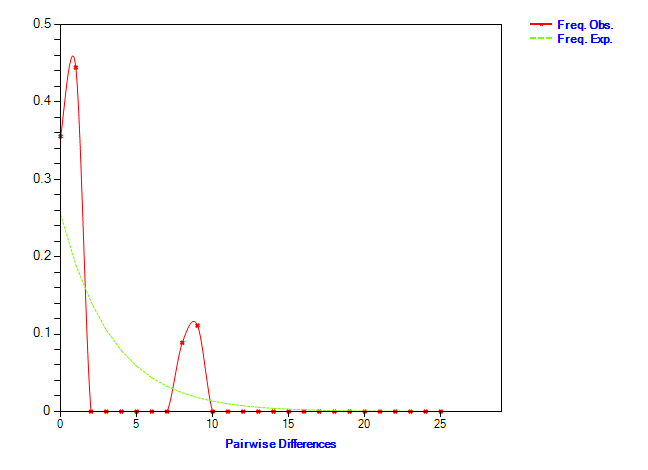

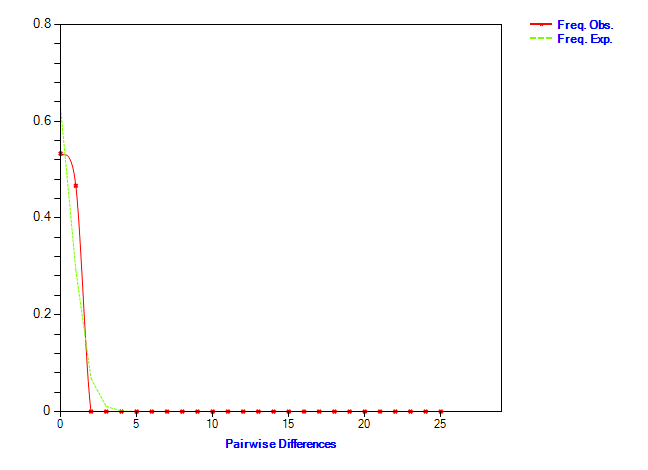


Negasi_Amba

Mihquan


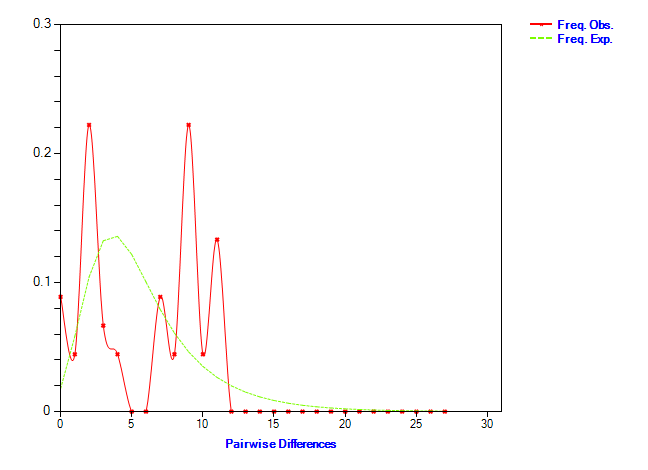

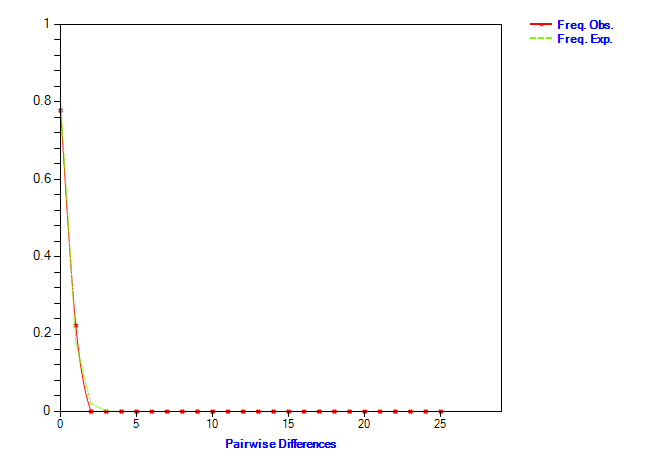


Shubi_Gemo

Surta


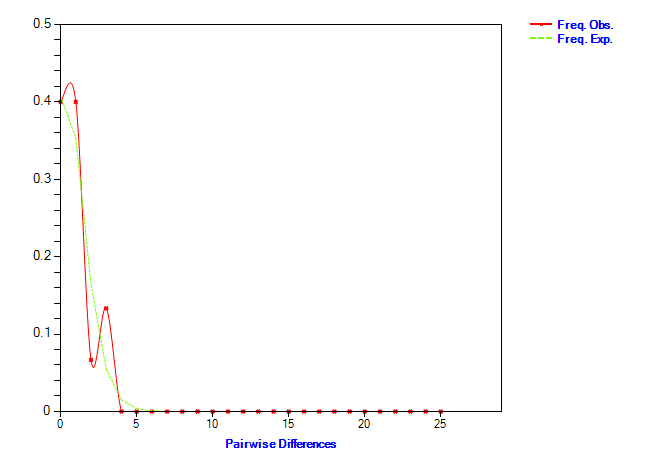


Tzion_Teguaz

**Fig. S10**

Table S1

| **Country** | **Accession no.** | **Reference** |
| --- | --- | --- |
| Chad | KT895348-KT895367 | Hassaballah *et al*. 2015 |
| Egypt | AB829473- AB829490 | Osman *et al*. 2016 |
| Nigeria | FJ851656-FJ851686 and GU951751-GU951758 | Adebambo *et al*. 2010 |
| Sudan | AM746042, AM746045 and AM746046 | Muchadeyi *et al*. 2008 |
| Turkey & Iran | KT596789- KT596807 | Meydan *et al*. 2016 |

Table S2a *P*-values above diagonal

|  | Baghdad | Karbala | Sulimania | Basra | Misan |
| --- | --- | --- | --- | --- | --- |
| Baghdad | --- | 0.949 | 0.001 | 0.003 | 0.024 |
| Karbala |  | --- | 0.006 | 0.021 | 0.122 |
| Sulimania |  |  | --- | 0.422 | 0.025 |
| Basra |  |  |  | --- | 0.139 |
| Misan |  |  |  |  | --- |

Table S2b *P*-values above diagonal

|  | Baghdad | Karbala | Sulimania | Basra | Misan |
| --- | --- | --- | --- | --- | --- |
| Baghdad | --- | 0.104 | 0.001 | 0.003 | 0.005 |
| Karbala |  | --- | 0.184 | 0.190 | 0.748 |
| Sulimania |  |  | --- | 0.910 | 0.228 |
| Basra |  |  |  | --- | 0.277 |
| Misan |  |  |  |  | --- |

Table S3a *P*-values above diagonal

|  | Mascara | Oran | Adrar | Tiaret | Tlemcen |
| --- | --- | --- | --- | --- | --- |
| Mascara | --- | 0.077 | 0.102 | 0.411 | 0.018 |
| Oran |  | --- | 0.810 | 0.024 | 0.664 |
| Adrar |  |  | --- | 0.037 | 0.458 |
| Tiaret |  |  |  | --- | 0.006 |
| Tlemcen |  |  |  |  | --- |

Table S3b *P*-values above diagonal

|  | Mascara | Oran | Adrar | Tiaret | Tlemcen |
| --- | --- | --- | --- | --- | --- |
| Mascara | --- | 0.492 | 0.140 | 0.976 | 0.001 |
| Oran |  | --- | 0.450 | 0.508 | 0.066 |
| Adrar |  |  | --- | 0.203 | 0.251 |
| Tiaret |  |  |  | --- | 0.031 |
| Tlemcen |  |  |  |  | --- |

Table S4a *P*-values above diagonal

|  | Shubi | Loya | Negasi | Batambie | Girissa | Gafera | Kumato | Horro | Tzion | Midir | Adane | Amshi | Ashuda | Dikuli | Arabo | Meseret | Mihquan | Jarso | Surta |
| --- | --- | --- | --- | --- | --- | --- | --- | --- | --- | --- | --- | --- | --- | --- | --- | --- | --- | --- | --- |
| Shubi | --- | 0.215 | 0.001 | 0 | 0.060 | 0.001 | 0.235 | 0.008 | 0.018 | 0.027 | 0.323 | 0.014 | 0 | 0.030 | 0.589 | 0.729 | 0.047 | 0 | 0 |
| Loya |  | --- | 0.030 | 0.001 | 0.452 | 0.035 | 1 | 0.247 | 0.182 | 0.199 | 0.643 | 0.054 | 0.003 | 0.207 | 0.454 | 0.303 | 0.305 | 0 | 0 |
| Negasi |  |  | --- | 0.124 | 0.135 | 1 | 0.029 | 0.068 | 0.243 | 0.224 | 0.005 | 0.612 | 0.304 | 0.239 | 0.004 | 0.003 | 0.179 | 0.001 | 0.059 |
| Batambie |  |  |  | --- | 0.010 | 0.112 | 0.003 | 0.006 | 0.025 | 0.021 | 0.001 | 0.048 | 0.392 | 0.022 | 0 | 0.001 | 0.011 | 0.056 | 0.834 |
| Girissa |  |  |  |  | --- | 0.144 | 0.455 | 0.819 | 0.576 | 0.607 | 0.217 | 0.223 | 0.022 | 0.638 | 0.177 | 0.101 | 0.877 | 0 | 0.004 |
| Gafera |  |  |  |  |  | --- | 0.034 | 0.080 | 0.230 | 0.252 | 0.013 | 0.596 | 0.307 | 0.235 | 0.007 | 0.007 | 0.169 | 0.002 | 0.066 |
| Kumato |  |  |  |  |  |  | --- | 0.258 | 0.204 | 0.221 | 0.635 | 0.073 | 0.007 | 0.226 | 0.454 | 0.319 | 0.322 | 0 | 0.001 |
| Horro |  |  |  |  |  |  |  | --- | 0.633 | 0.636 | 0.092 | 0.231 | 0.008 | 0.642 | 0.043 | 0.030 | 0.993 | 0 | 0.003 |
| Tzion |  |  |  |  |  |  |  |  | --- | 1 | 0.105 | 0.531 | 0.046 | 1 | 0.040 | 0.046 | 0.755 | 0 | 0.007 |
| Midir |  |  |  |  |  |  |  |  |  | --- | 0.127 | 0.524 | 0.043 | 1 | 0.049 | 0.051 | 0.759 | 0 | 0.008 |
| Adane |  |  |  |  |  |  |  |  |  |  | --- | 0.035 | 0.001 | 0.123 | 0.752 | 0.618 | 0.205 | 0 | 0 |
| Amshi |  |  |  |  |  |  |  |  |  |  |  | --- | 0.142 | 0.543 | 0.016 | 0.013 | 0.309 | 0.001 | 0.016 |
| Ashuda |  |  |  |  |  |  |  |  |  |  |  |  | --- | 0.045 | 0 | 0 | 0.035 | 0.008 | 0.257 |
| Dikuli |  |  |  |  |  |  |  |  |  |  |  |  |  | --- | 0.052 | 0.045 | 0.776 | 0.001 | 0.010 |
| Arabo |  |  |  |  |  |  |  |  |  |  |  |  |  |  | --- | 0.877 | 0.096 | 0 | 0 |
| Meseret |  |  |  |  |  |  |  |  |  |  |  |  |  |  |  | --- | 0.088 | 0 | 0 |
| Mihquan |  |  |  |  |  |  |  |  |  |  |  |  |  |  |  |  | --- | 0 | 0.003 |
| Jarso |  |  |  |  |  |  |  |  |  |  |  |  |  |  |  |  |  | --- | 0.055 |
| Surta |  |  |  |  |  |  |  |  |  |  |  |  |  |  |  |  |  |  | --- |

Table S4b *P*-values above diagonal

|  | Shubi | Loya | Negasi | Batambie | Girissa | Gafera | Kumato | Horro | Tzion | Midir | Adane | Amshi | Ashuda | Dikuli | Arabo | Meseret | Mihquan | Jarso | Surta |
| --- | --- | --- | --- | --- | --- | --- | --- | --- | --- | --- | --- | --- | --- | --- | --- | --- | --- | --- | --- |
| Shubi | --- | 0.219 | 0 | 0.001 | 0.005 | 0.001 | 0.167 | 0.032 | 0.007 | 0.003 | 0.164 | 0 | 0.001 | 0.002 | 0.012 | 0.107 | 0.051 | 0 | 0 |
| Loya |  | --- | 0.093 | 0.085 | 0.175 | 0.092 | 0.864 | 0.580 | 0.161 | 0.121 | 0.869 | 0.117 | 0.085 | 0.113 | 0.227 | 0.691 | 0.481 | 0.035 | 0.074 |
| Negasi |  |  | --- | 0.916 | 0.714 | 1 | 0.134 | 0.119 | 0.792 | 0.911 | 0.105 | 0.971 | 0.969 | 0.922 | 0.628 | 0.180 | 0.375 | 0.791 | 0.891 |
| Batambie |  |  |  | --- | 0.684 | 0.920 | 0.106 | 0.130 | 0.715 | 0.859 | 0.125 | 0.871 | 0.962 | 0.829 | 0.602 | 0.185 | 0.327 | 0.892 | 0.989 |
| Girissa |  |  |  |  | --- | 0.727 | 0.256 | 0.265 | 0.937 | 0.829 | 0.251 | 0.761 | 0.685 | 0.820 | 0.906 | 0.337 | 0.553 | 0.524 | 0.655 |
| Gafera |  |  |  |  |  | --- | 0.119 | 0.133 | 0.811 | 0.918 | 0.126 | 0.963 | 0.956 | 0.902 | 0.674 | 0.186 | 0.369 | 0.784 | 0.897 |
| Kumato |  |  |  |  |  |  | --- | 0.724 | 0.186 | 0.128 | 0.999 | 0.140 | 0.121 | 0.147 | 0.299 | 0.822 | 0.580 | 0.053 | 0.096 |
| Horro |  |  |  |  |  |  |  | --- | 0.216 | 0.163 | 0.717 | 0.133 | 0.117 | 0.165 | 0.352 | 0.918 | 0.723 | 0.039 | 0.105 |
| Tzion |  |  |  |  |  |  |  |  | --- | 0.862 | 0.187 | 0.837 | 0.752 | 0.878 | 0.848 | 0.285 | 0.504 | 0.587 | 0.685 |
| Midir |  |  |  |  |  |  |  |  |  | --- | 0.155 | 0.949 | 0.840 | 1 | 0.739 | 0.251 | 0.421 | 0.682 | 0.805 |
| Adane |  |  |  |  |  |  |  |  |  |  | --- | 0.142 | 0.109 | 0.144 | 0.296 | 0.838 | 0.553 | 0.051 | 0.099 |
| Amshi |  |  |  |  |  |  |  |  |  |  |  | --- | 0.930 | 0.943 | 0.695 | 0.200 | 0.373 | 0.753 | 0.872 |
| Ashuda |  |  |  |  |  |  |  |  |  |  |  |  | --- | 0.861 | 0.607 | 0.178 | 0.340 | 0.808 | 0.948 |
| Dikuli |  |  |  |  |  |  |  |  |  |  |  |  |  | --- | 0.736 | 0.218 | 0.427 | 0.674 | 0.803 |
| Arabo |  |  |  |  |  |  |  |  |  |  |  |  |  |  | --- | 0.423 | 0.645 | 0.454 | 0.561 |
| Meseret |  |  |  |  |  |  |  |  |  |  |  |  |  |  |  | --- | 0.713 | 0.082 | 0.145 |
| Mihquan |  |  |  |  |  |  |  |  |  |  |  |  |  |  |  |  | --- | 0.187 | 0.314 |
| Jarso |  |  |  |  |  |  |  |  |  |  |  |  |  |  |  |  |  | --- | 0.882 |
| Surta |  |  |  |  |  |  |  |  |  |  |  |  |  |  |  |  |  |  | --- |

Table S5a *P*-values above diagonal

|  | East | Central | West |
| --- | --- | --- | --- |
| East | --- | 0.001 | 0.062 |
| Central |  | --- | 0.040 |
| West |  |  | --- |

Table S5b *P*-values above diagonal

|  | East | Central | West |
| --- | --- | --- | --- |
| East | --- | 0.537 | 0.972 |
| Central |  | --- | 0.477 |
| West |  |  | --- |

| **Country/population** | **N** | **S** | **H** | **Hd (SD)** | **𝜋 (SD)** | **K** |
| --- | --- | --- | --- | --- | --- | --- |
| Algeria_Haplogroup E | 87 | 12 | 12 | 0.588(0.060) | 0.0024(0.0003) | 0.955 |
| Ethiopia_Haplogroup E | 169 | 25 | 27 | 0.770(0.023) | 0.0040(0.0003) | 1.613 |
| Iraq_Haplogroup E | 84 | 13 | 11 | 0.512(0.064) | 0.0032(0.0005) | 1.299 |
| Libya_Haplogroup E | 21 | 8 | 8 | 0.676(0.111) | 0.0025(0.0006) | 1.010 |
| Pakistan_Haplogroup E | 84 | 19 | 17 | 0.717(0.048) | 0.0062(0.0005) | 2.485 |
| Saudi_Haplogroup E | 180 | 24 | 23 | 0.712(0.034) | 0.0032(0.0003) | 1.273 |

Table S6
